# Association of Serum Organophosphorus Pesticide Levels with T2D Risk and Blood Glucose Changes: A Nested Case–Control Study

**DOI:** 10.3390/toxics14040283

**Published:** 2026-03-26

**Authors:** Yan Yan, Chengyong Jia, Xu Cheng, Jun An, Peiwen Li, Jiazhen Zhang, Weiya Li, Meian He

**Affiliations:** 1 Department of Occupational and Environmental Health, School of Public Health, Tongji Medical College, Huazhong University of Science and Technology, 13 Hangkong Rd., Wuhan 430030, China; 2 Ministry of Education Key Laboratory of Environment and Health, State Key Laboratory of Environmental Health (Incubating), School of Public Health, Tongji Medical College, Huazhong University of Science and Technology, 13 Hangkong Rd., Wuhan 430030, China

**Keywords:** organophosphorus pesticides, fasting blood glucose, type 2 diabetes, prospective study

## Abstract

Organophosphorus pesticides (OPs) are widely used in agriculture, but prospective studies on their chronic exposure and risk of type 2 diabetes (T2D) and glucose metabolism disorders are scarce. Most previous studies focused on agricultural workers and relied on questionnaires or urinary metabolites for exposure assessment. We conducted a nested case–control study with 1006 pairs of participants based on the Dongfeng–Tongji cohort to investigate the association between serum OP levels, T2D risk, and fasting blood glucose (FBG) changes over a 5-year follow-up. Serum OP concentrations were measured by gas chromatography–triple quadrupole mass spectrometry. Among the 29 types of OPs detected, Chlorpyrifos and Fenitrothion had detection rates of 99.9% and 87.9%, respectively. Etrimfos and Parathion were detected in 75.8% and 64.5% of participants. Four types of OPs—Ethoprophos, Phorate, Diazinon, and Malathion, categorized into ≤LOD and >LOD groups—had detection rates ranging from 20% to 60%. OP exposure was not associated with T2D risk in the overall population. Among participants with baseline FBG ≥ 6.1 mmol/L, OP exposure showed a positive association with incident T2D and with increases in FBG during a 5-year follow-up. In contrast, OP exposure was associated with decreased FBG in the overall population. Moreover, significant interactions were observed between OP exposure and baseline FBG levels (*P_interaction_* < 0.05), suggesting that baseline glucose levels may modify the metabolic effects of chronic OP exposure. These findings highlight the importance of considering basal glucose status when evaluating the long-term metabolic effects of OP exposure.

## 1. Introduction

As one of the top 10 leading causes of death worldwide [1], type 2 diabetes (T2D) has caused serious disease threats, strained medical resources, and economic burdens in both developed and developing countries [2]. The estimated number of adults suffering from diabetes reached 540 million in 2021 globally [3], among which T2D accounted for more than 90% [4]. Worse still, researchers have estimated that the number will reach 552 million by 2030 [5]. In China, the prevalence rate has increased from 0.67% in 1980 to 12.8% in 2017 [6]. It is universally recognized that age, lifestyle, genetic susceptibility, and environmental pollutant exposure are identified as risk factors for T2D [7]. In recent years, an increasing number of studies have explored whether environmental chemicals, including persistent organic pollutants (POPs), heavy metals such as arsenic and cadmium, and certain endocrine-disrupting chemicals like bisphenol A and phthalates, are associated with T2D [8]. In particular, the combined effect may exacerbate the process.

Pesticides are widely used for agricultural purposes, and China is one of the world’s largest pesticide producers and consumers. The annual application amount of pesticides in China was about 1.8 million tons in 2019, while only about 380,000 tons was used in the United States [9]. Organophosphorus pesticides (OPs) are phosphoric acid or phosphorothioate substances with a wide insecticidal spectrum and high insecticidal efficiency. OPs mainly remain in the soil, the water environment, and crops due to pesticide spraying application, manufacturing, transportation, trafficking, and cleaning residues, and they enter the human body through ingestion, inhalation, or skin contact.

Neurotoxicity is the most well-known and extensively studied characteristic of OPs, manifesting as muscarinic and nicotinic effects on the nervous system during acute poisoning. However, beyond these neurological effects, accumulating evidence indicates that OP exposure can also disrupt glucose metabolism and may contribute to the development of diabetes. A previous clinical case found that patients with acute organophosphorus pesticide poisoning presented with diabetic ketoacidosis [10]. Acute organophosphorus pesticide poisoning has also been found to lead to hyperglycemic status or elevated insulin [11]. Some animal studies demonstrated that acute or subacute chronic OP exposure led to hyperglycemia in mice [12]. Moreover, chlorpyrifos exposure during pregnancy resulted in insulin resistance, hyperglycemia, and hyperlipidemia in female rats and offspring [13]. Some zoological studies revealed that OP exposure was not associated with decreased blood glucose in aquatic animals or mammals [14,15]. Exposure to pesticides in agricultural occupational conditions has been shown to be associated with an increased risk of diabetes [16]. The Agricultural Health Study (AHS) was a large prospective cohort study of agricultural workers and their spouses in North Carolina and Iowa. The health effects of exposure to pesticides were assessed by investigating the pesticides they had ever used and the cumulative lifetime days of use [17]. A prospective study based on the AHS involving more than 30,000 people found that 7 of the 10 organophosphorus pesticides were associated with an increased risk of diabetes, 3 of which showed a dose–response relationship [18]. A case–control study in China found that participants with a history of chronic exposure to OPs were 1.836 times more likely to develop T2D than those without chronic exposure [19]. However, a study in Iran showed that exposure to pesticides was not associated with the prevalence of T2D [20]. In addition, the longitudinal effect of chronic OP exposure on blood glucose is still unknown. In existing mechanistic studies, oxidative stress, lipid peroxidation, and inflammatory responses have been found to impair pancreatic β-cell function, thereby contributing to insulin resistance and the development of metabolic syndrome [21]. In addition, organophosphorus pesticides may interfere with key enzymes involved in glucose metabolism, for example, glucose-6-phosphatase, a terminal rate-limiting enzyme in gluconeogenesis and glycogenolysis, whose dysregulation can lead to the onset of insulin resistance [22]. More studies are needed to investigate the effect of OP exposure on glucose homeostasis. In addition, because most existing studies aimed at agricultural occupational groups, studies need to be carried out in other population with less exposure to pesticides. To find out whether long-term chronic exposure to OPs causes disturbances in the blood glucose level, we investigated the association between serum levels of multiple organophosphorus pesticides and new-onset T2D and changes in blood glucose in non-agricultural occupational groups in China.

## 2. Methods

### 2.1. Study Population

The Dongfeng–Tongji cohort was established in 2008 to investigate the effects of various risk factors on the development of chronic diseases [23]. A total of 27,009 retired employees of Dongfeng Motor Corporation (DMC) were included at baseline, and their basic information and biological samples were collected. The present study was designed as a nested case–control study within this prospective cohort. Inclusion and exclusion criteria are shown in Figure S1. Participants were followed from 2008 to 2013 to ascertain incident T2D. Fasting blood samples were collected at baseline in 2008 and at follow-up visit in 2013. For each incident T2D case, one control was selected from participants who remained free of T2D during the follow-up period and met the eligibility criteria. Controls were individually matched to cases by sex and age (±5 years) after excluding those with diabetes, cardiovascular disease, cancer, or serum samples that were insufficient to measure organophosphorus pesticide concentrations. A total of 1006 pairs of cases and controls were included in the final analysis.

### 2.2. Determination of Serum Organophosphorus Pesticide Concentrations

Baseline fasting blood samples collected in 2008 were used for measurement of serum organophosphorus pesticide concentrations. Referring to a previous study [24], fasting blood samples were collected at baseline in coagulation vessels and stored in a −80 °C freezer until serum OP concentrations were measured from May 2021 to August 2021. To minimize potential batch effects during laboratory analysis, matched case–control serum sample pairs were randomly assigned to analytical batches and analyzed within the same batch. The study referred to the previously established detection method for multiple pollutants in serum [25]. Serum samples were spiked with an internal standard solution (10 ng/mL), mixed with ultrapure water and acetonitrile (Anpel Laboratory Technologies Inc., Shanghai, China), and subjected to ultrasonic extraction. The mixture was extracted with n-hexane followed by n-hexane/dichloromethane (1:1, *v*/*v*), vortexed, and centrifuged at 3000 r/min for 10 min at room temperature. The combined organic phase was evaporated under a nitrogen stream at 35 °C and reconstituted in n-hexane prior to GC–MS analysis. Blank samples consisted of fetal bovine serum (FBS; Gibco, Thermo Fisher Scientific, Waltham, MA, USA) processed using the same extraction procedure to monitor potential contamination. Standard solutions of OPs (100 μg/mL in toluene; Alta Scientific Co., Tianjin, China) were used to prepare calibration standards. Mixed standard working solutions were prepared in n-hexane and stored at 4 °C in the dark. Matrix-matched calibration curves were constructed by spiking pre-extracted FBS matrix with mixed standards to obtain concentrations of 0.1–200 ng/mL. All calibration samples contained internal standard solution (10 ng/mL). We used gas chromatography (Agilent 8890 gas chromatography system; Agilent Technologies, Clara, CA, USA) with a DB-5 capillary column to separate organophosphorus pesticides, followed by tandem triple quadrupole mass spectrometry (Agilent 7010B mass spectrometry; Agilent Technologies, Clara, CA, USA) for multiple reaction monitoring and quantification of organophosphorus pesticide concentrations. For quality control, pooled serum samples were prepared by combining equal aliquots from 150 randomly selected samples, which were processed and analyzed together with each analytical batch to monitor analytical stability and reproducibility. The limit of detection (LOD), spike recovery, and precision of the analytical method are summarized in Supplementary Table S1.

In the present study, 4 types of OPs, including Etrimfos, Chlorpyrifos, Fenitrothion and Parathion, with detection rates ≥ 64.5%, were included, and concentrations lower than the LOD were replaced by LOD divided by √2 [26]. ΣOrganophosphorus pesticides was derived from the addition of the 4 OP concentrations above. Substances with an LOD between 20% and 60% were divided into two groups of an undetected group and a detected group for subsequent analysis.

### 2.3. Ascertainment of Type 2 Diabetes

According to the American Diabetes Association, incident T2D cases were ascertained as self-reporting of a physician diagnosis of diabetes, taking oral hypoglycemic agents or insulin medications, or measuring a fasting blood glucose level ≥ 7.0 mmol/L or glycosylated hemoglobin level ≥ 6.5% during the follow-up period [27].

### 2.4. Covariates

We obtained the general characteristics of the participants by epidemiological questionnaires when establishing baseline files, such as gender, age, education level, marital status, physical activity, smoking and drinking status, family history of diabetes mellitus, and hypoglycemic drug use. Self-reported educational level was defined as primary school or below, junior high school, senior high school or secondary school, college or above. Smoking and drinking status were categorized into never, ever, and present. Physical activity was defined as regular exercise for more than 20 min per session for at least half a year.

Trained operators measured height, weight, and blood pressure of participants at baseline. Body mass index (BMI) was equal to participants’ weight (kg) divided by the square of their height (m^2^). According to the current criteria for adult weight in China, BMI ≥ 24 kg/m^2^ was defined as overweight and obese. Hypertension was defined as systolic blood pressure ≥ 140 mmHg or diastolic blood pressure ≥ 90 mmHg or self-reported oral antihypertensive agents or physician diagnosis of hypertension. We used an ARCHITECT Ci8200 automated analyzer (ABBOTT Laboratories, Abbott Park, IL, USA) to measure total cholesterol (TC), triglycerides (TG), high-density lipoprotein (HDLc), low-density lipoprotein (LDLc), and fasting blood glucose (FBG) in the laboratory of DMC hospital. Hyperlipidemia was defined as total cholesterol (TC) ≥ 5.72 mmo1/L, triglycerides (TG) ≥ 1.17 mmo1/L or clinical diagnosis of hyperlipidemia or taking lipid-lowering drugs. The estimated glomerular filtration rate (eGFR) was calculated by the formula published in a previous study [28].

### 2.5. Statistical Analyses

We divided the total population into four subgroups according to quartiles of ΣOrganophosphorus pesticides concentrations. For basic characteristics of subgroups, the Kruskal–Wallis test was used for continuous variables and chi-square test for categorical variables. Because of the skewed distribution characteristics, we used Spearman’s rank correlation to investigate the association of ln-transformed organophosphorus pesticides levels.

The odds ratio (OR) and 95% confidence interval (95% CI) of T2D were evaluated by the conditional logistic regression model. Serum organophosphorus pesticide levels were included in the model after ln-transformation and quartile classification. The trend test was performed by including the median of the OP quartiles as continuous variables in the model. Model 1 adjusted for age; model 2 further adjusted for BMI, education level, smoking status, drinking status, physical activity, family history of diabetes mellitus, hypertension, and hyperlipidemia.

Blood glucose change was defined as the FBG value at follow-up minus the baseline, and the blood glucose change rate was obtained by dividing the absolute blood glucose changes over a 5-year follow-up by the baseline FBG. Generalized linear models were used to investigate the association between ln-transformed OP levels and blood glucose changes/blood glucose change rate over a 5-year follow-up. Model 1 adjusted for age and gender; model 2 additionally adjusted for smoking status, drinking status, education level, BMI, physical activity, family history of diabetes, and hypoglycemic drugs used in the past two weeks. To eliminate the effect of hypoglycemic drug use on glycemic changes, we performed a sensitivity analysis excluding participants who had used hypoglycemic drugs in the past 2 weeks at follow-up.

Data analysis for this study used the IBM SPSS Statistics (version 26.0; IBM Corp., Armonk, NY, USA) and R software (version 4.2.0; R Foundation for Statistical Computing, Vienna, Austria). A two-side *p* value < 0.05 was considered to be statistically significant.

## 3. Results

### 3.1. Baseline Characteristics

Baseline demographic characteristics of 2012 participants are presented in Table 1. As the ΣOrganophosphorus pesticides concentration increased, males accounted for a higher proportion. The ΣOPs concentration was 1071.8 (754.9, 1461.6) ng/L in males and 915.9 (648.9, 1316.9) ng/L in females. Significant differences were also observed across groups in smoking and drinking status, physical activity, baseline FBG, and HDLc levels. However, other characteristics were not statistically different among the ΣOrganophosphorus pesticides quartiles.

Table 2 summarizes the types of organophosphorus pesticides detected in the present study and their detection rates. There were two types of serum OPs, Chlorpyrifos and Fenitrothion, with detection rates of 99.9% and 87.9%, respectively. Etrimfos had a detection rate of 75.8%, and Parathion had a detection rate of 64.5%, among which there was no significant difference in baseline concentrations between the cases and the controls (Table 3). The remaining four types of serum OPs (Ethoprophos, Phorate, Diazinon, and Malathion) had detection rates ranging from 20% to 60% and were categorized into ≤LOD and >LOD groups. As shown in Figure S2, Spearman’s rank coefficients among serum Etrimfos, Chlorpyrifos, Fenitrothion, and Parathion levels were −0.07–0.58.

Continuous variables, presented as medians (25th–75th percentile), were compared with the Kruskal–Wallis test, while categorical variables, presented as N (%), were tested by the chi-square test.

### 3.2. Association Between Serum Organophosphorus Pesticides Levels and Risk of T2D

Table 4 presents the association between serum OP levels and risk of T2D. None of the OP levels were significantly associated with the risk of T2D. We further conducted a stratified analysis based on baseline blood glucose. The results showed that in the group with baseline blood glucose ≥ 6.1 mmol/L, serum OP levels were positively correlated to risk of T2D in the second and third quartiles, but in those with baseline blood glucose < 6.1 mmol/L, the association was negative without statistical significance (Supplementary Table S2). We also stratified the participants by gender, smoking and drinking status, physical activity, and HDLc levels. According to the established clinical reference criteria for cardiovascular risk stratification (MSD Professional Edition Manual), normal HDL-C levels were defined as ≥1.0 mmol/L in men and ≥1.3 mmol/L in women. In any subgroup, OPs were not significantly associated with the risk of new-onset type 2 diabetes (Supplementary Tables S3–S7). Among the four types of OPs with an LOD between 20% and 60% (Ethoprophos, Phorate, Diazinon, and Malathion), there was no significant association between OPs and risk of T2D (Supplementary Table S8).

### 3.3. Association Between Serum Organophosphorus Pesticide Levels and Blood Glucose Changes/Blood Glucose Change Rate

**Figure 1:**
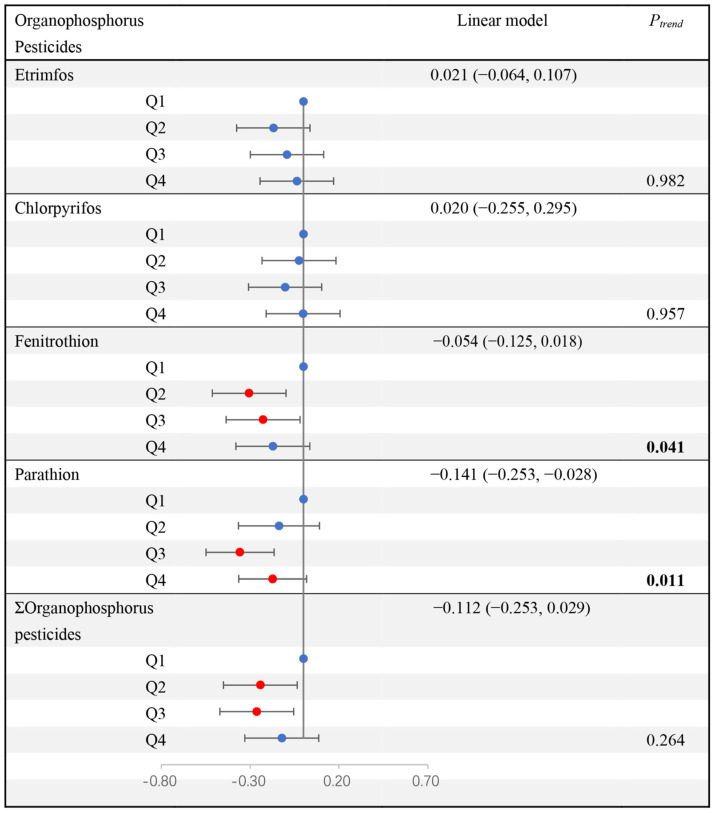
Association between serum organophosphorus pesticides (OPs) and blood glucose changes during the 5-year follow-up. *P_trend_* was obtained from the median of each quartile (ln-transformed) in the generalized linear regression model as a continuous variable. Linear model: each ln-transformed concentration was included in the generalized linear regression model as a continuous variable, adjusting for age, gender, smoking status, drinking status, education level, BMI, physical activity, family history of diabetes mellitus, and hypoglycemic drugs. Blue dots represented non-significant results, red dots represented statistically significant results.

After a 5-year follow-up, blood glucose levels in the study population ranged from 5.70 (5.30, 6.20) at baseline to 6.10 (5.40, 7.20) mmol/L. As shown in Figure 1, we found that compared with the reference group, the second and third quartiles of Fenitrothion and ΣOrganophosphorus pesticides were significantly negatively correlated with blood glucose changes. Compared to the first quartile, the β-values (95% CI) for changes in blood glucose levels in the second and third quartiles of Fenitrothion were −0.306 (−0.514, −0.099) and −0.227 (−0.435, −0.019), respectively (*P_trend_* = 0.041). For ΣOrganophosphorus pesticides, the corresponding β-values of the second and third quartiles were −0.242 (−0.449, −0.034) and −0.262 (−0.469, −0.054) respectively after adjustment for covariates. In addition, we discovered that there was a significant negative correlation between serum Parathion levels and blood glucose level changes. Each ln-Parathion increase was associated with a −0.141 (−0.253, −0.028) mmol/L blood glucose level change (*P_trend_* = 0.011). We found similar negative associations with the change rate of blood glucose levels during the 5-year follow-up period.

**Figure 2:**
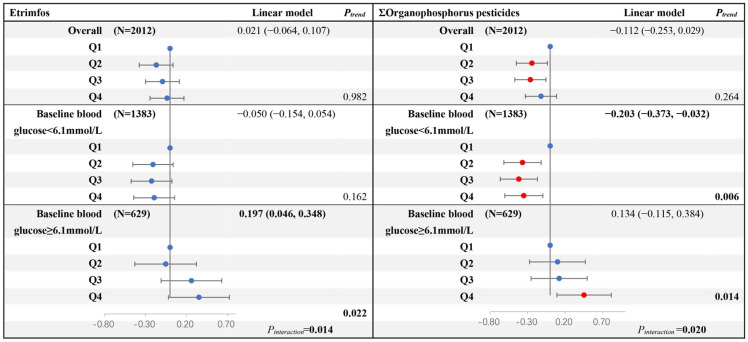
Stratified analysis for the association between serum Etrimfos/OPs and blood glucose changes during the 5-year follow-up. *P_trend_* was obtained from the median of each quartile (ln-transformed) in the generalized linear regression model as a continuous variable. Linear model: each ln-transformed concentration was included in the generalized linear regression model as a continuous variable, adjusting for age, gender, smoking status, drinking status, education level, BMI, physical activity, family history of diabetes mellitus, and hypoglycemic drugs. Blue dots represented non-significant results, red dots represented statistically significant results.

We then performed a subgroup analysis by dividing baseline blood glucose into <6.1 mmol/L and ≥6.1 mmol/L group. In the subgroup with baseline FBG ≥ 6.1 mmol/L, Etrimfos and ΣOrganophosphorus pesticides were positively associated with blood glucose changes, with changes of 0.197 (0.046, 0.348) and 0.134 (−0.115, 0.384) for each ln-increase, respectively (Figure 2). In the subgroup with baseline FBG < 6.1 mmol/L, ΣOrganophosphorus pesticides were significantly negatively associated with blood glucose changes, with a change of −0.203 (−0.373, −0.032) per ln-increase (*P_trend_* = 0.006). A negative association was also observed for Etrimfos. In addition, we identified a significant interaction between Etrimfos/ΣOrganophosphorus pesticides and baseline FBG levels on blood glucose changes, with *P_interaction_* of 0.014 and 0.020, respectively, and similar results were obtained for glucose change rates (*P_interaction_* = 0.022 and 0.020, respectively) (Figures S3–S7). Baseline FBG status significantly modified the relationship between organophosphorus pesticide levels and blood glucose changes, suggesting that basal glucose levels might be an important effect modifier. In the subgroup stratified by gender, smoking, drinking status, physical activity, and HDLc levels, similar findings were observed, consistent with those in the total participants. No significant interaction effects were detected (*P_interaction_* > 0.05) (Supplementary Tables S9–S13).

After exclusion of those with hypoglycemic drug use, similar results were obtained (Supplementary Table S14). Among those with detection rates between 20% to 60%, a negative correlation between Phorate, Diazinon and blood glucose changes was found, with β-values (95% CI) of −0.216 (−0.363, −0.068) and −0.196 (−0.369, −0.023) respectively (Supplementary Table S15), and similar results were found for the blood glucose change rate (Supplementary Table S16).

## 4. Discussion

In the present nested case–control study, we did not find an association between OPs and the risk of T2D. However, in individuals with baseline FBG ≥ 6.1 mmol/L, higher OP exposure was significantly associated with an increased risk of T2D when comparing with the lowest quartile. Moreover, among participants with elevated baseline FBG, Etrimfos and ΣOrganophosphorus pesticides were positively associated with blood glucose changes over the 5-year follow-up; on the contrary, in those with baseline FBG < 6.1 mmol/L, Etrimfos and ΣOrganophosphorus pesticides were negatively associated with blood glucose changes, consistent with the total participants. Stratifying the analyses by sex and lifestyle-related risk factors, including smoking status, drinking status, physical activity, and HDL-C levels, did not find significant interaction on FPG changes between OP exposure and these factors. Notably, baseline glycemic status showed a significant interaction effect, which suggested that OP exposure might primarily affect individuals with pre-existing metabolic vulnerability and that individuals with impaired glucose regulation might be more susceptible to the potential metabolic effects of OPs. The present study is the first to reveal the important role of baseline blood glucose levels in the association of organophosphorus pesticides with blood glucose metabolism. The finding not only enhances our understanding of the mechanisms underlying environmental toxicants but also offers new insights for early screening and targeted interventions in high-risk populations.

In cotton-growing areas, plasma Malathion and Chlorpyrifos concentrations of pesticides applicators were 177.53 ± 99.74 ng/mL and 1.09 ± 0.44 ng/mL in Pakistan and 48.80 ± 30.12 ng/mL and 1.44 ± 0.98 ng/mL in Cameroon [29]. Serum OP concentrations measured by GC-MS of 140 Indian women were 2556 ± 1027 ug/L of Malathion and 1036 ± 1413 ug/L of Chlorpyrifos in spontaneous abortion cases and 1253.7 ± 1421 ug/L of Malathion and 106.3 ± 129 ug/L of Chlorpyrifos respectively in controls [30]. Obviously, OP concentrations detected in the present study population were lower than those in pesticides application areas. To date, the majority of studies on pesticide health effects have focused on agricultural workers, but they could not delineate exposure to pesticides of the general population daily. In fact, pesticide residues have been detected in fruits and vegetables sold by supermarkets [31]. Therefore, it is important to explore pesticide residues in non-occupationally exposed populations.

An accumulating number of studies have suggested that long-term and chronic OP exposure led to the development of metabolic syndromes, such as obesity, hyperlipidemia, and hyperglycemia [32]. A case–control study of Thai residents showed that Mevinphos use was significantly associated with an increased odds ratio of diabetes [16]. The AHS cohort demonstrated that use of specific OPs led to an increased risk of diabetes in farmers’ wives, where HRs for Fonofos, Phorate, and Parathion were 1.56 (95% CI: 1.11–2.19), 1.57 (95% CI: 1.14–2.16), and 1.61 (95% CI: 1.05–2.46), respectively [33]. Agricultural workers had higher levels of FBG as well as oral glucose tolerance test (OGTT) results than the control group in a cross-sectional study [34]. In an Indian population (n = 3080), plasma OP levels were positively correlated with the prevalence of diabetes [35]. In a study of NHANSE adults over 20 years old, six organophosphorus pesticide metabolites in urine were positively associated with diabetes risk [36]. Anne Debost-Legrand et al. found a potential association between urinary metabolites of OPs in the first trimester and altered glucose metabolism at birth [37]. A case–control study carried out in a rural population in Henan Province, China, found that plasma OP levels were significantly and positively associated with the risk of T2D and positively associated with FBG and glycosylated hemoglobin levels [38]. The team’s latest study further supported that exposure to pesticides alone and in combination increased the incidence of T2D in the impaired fasting glucose (IFG) population [39].

Conversely, a cross-sectional study carried out in Iran investigating pesticide use among 9088 study subjects did not find an association between pesticides and T2D prevalence [20], which was consistent with the present findings in the overall population. Our study found a negative association between OP exposure and longitudinal blood glucose changes over 5 years in the general population, differing from most previous studies. Stratifying participants by baseline FBG levels, we found that in participants with lower baseline FBG (<6.1 mmol/L), blood glucose decreased with increasing OP levels after a 5-year follow-up, which suggested that normoglycemic individuals might be more sensitive to OP toxicity, possibly exhibiting lower metabolic rates or relatively excessive insulin secretion in response to exposure to relatively low OPs levels, resulting in reduced fasting glucose changes. Conversely, in participants with higher baseline FBG (≥6.1 mmol/L), OP levels were positively associated with increased blood glucose levels, indicating a potential exacerbation of metabolic dysfunction. This observation aligned with an increased trend in T2D risk in individuals with high baseline glucose, indicating that individuals with hyperglycemia might be more susceptible to the adverse metabolic effects of OPs. Such a discrepancy suggested that the effect of OP exposure on glucose metabolism may vary depending on individual metabolic conditions, particularly baseline blood glucose levels. Our cross-sectional analysis also revealed a positive association between OP exposure and baseline FBG levels (Supplementary Table S17), supporting the hypothesis that OPs might contribute to elevated basal blood glucose through long-term effects. In individuals with normoglycemia at baseline, OP exposure might lead to minor fluctuations in glucose metabolism in the short term, but these changes did not appear to accumulate over time. In contrast, in those with pre-existing hyperglycemia, OP exposure might aggravate insulin resistance, leading to a sustained rise in blood glucose levels. This potential interaction between OP exposure and baseline glycemic status partly explained the inconsistencies observed across previous epidemiological studies.

Experimental studies provided additional support for OP-induced glycemic disturbances [40]. Researchers established animal models to investigate the effect of OPs on glucose metabolism disorders. After acute Chlorpyrifos exposure in rats, Acker, C.I et al. indicated that Chlorpyrifos caused increases in glucose, lipid level, and glucose-6-phosphatase (G-6-pase) activity [41]. A systematic review revealed that Chlorpyrifos led to hyperglycemia, hyperlipidemia, and decreased insulin levels in rodents or fish; in addition, such a metabolic change was time-dependent [42]. Findings regarding specific OP compounds remained inconsistent. While some studies reported increased blood glucose in rats exposed to Fenitrothion and Parathion [43,44,45], others found no effect of Fenitrothion on blood glucose in mice after 28 days of exposure [15]. Freshwater fish exposed to Parathion for 96 h were found to have 55% lower plasma glucose levels [14]. In the present study, despite the lack of an observed effect of serum Chlorpyrifos on glucose metabolism, we found that Fenitrothion, Parathion, and total OP levels were negatively associated with blood glucose changes over 5 years in the total population. Among compounds with moderate detection rates (20–60%), Phorate and Diazinon were also negatively associated with blood glucose changes. These findings pointed out that the metabolic effects of OPs differed across compounds and exposure levels. Furthermore, the observed positive association between OP levels and blood glucose changes in individuals with baseline hyperglycemia highlighted a potential susceptibility among individuals with pre-existing metabolic dysfunction.

Inhibition of acetylcholinesterase activity by OPs has been demonstrated in zoological studies and epidemiological studies [46]. Organophosphorus phosphate may also damage pancreatic β-cells through oxidative damage and lipid peroxidation, inducing obesity, insulin resistance, and the development of metabolic syndrome [21]. It may also cause cell damage due to the action of pro-inflammatory factors; for instance, Chlorpyrifos promoted apoptosis by up-regulating the expression of pro-inflammatory genes *TNF-a*, *IL-8*, and *IL-15*, down-regulating the expression of anti-inflammatory genes *TGF-β1* and *IL-10*, and up-regulating the expression of *caspase-3*, *caspase-8*, *caspase-9*, and *Bax* [22]. OPs disturbed glucose homeostasis and increased the incidence of metabolic disorders and diabetes through insulin resistance [47]. Animal studies attempted to reveal the molecular mechanisms underlying the hyperglycemic status induced by OP exposure. Chlorpyrifos has been found to interfere with enzymes of blood glucose metabolism, including glucose-6-phosphatase (G-6-Pase), a key enzyme that induces hepatic gluconeogenesis [41]. Although most studies revealed the effect of OP exposure on blood glucose elevation in animals, some have yielded contrasting results [14,15]. The underlying mechanisms of blood glucose disturbances caused by organophosphorus pesticides need to be confirmed in further studies.

The advantage of this study was that the relationship between pesticide exposure and T2D risk and glycemic variability was prospectively analyzed. In addition, instead of using a traditional questionnaire or urine metabolites to assess OP exposure levels, the present study measured serum OP levels by GC-MS, which could relatively reduce exposure misclassification and investigate the longitudinal health effects of these trace amounts of contaminants remaining in the human body prototype. More importantly, the present study was the first to reveal that individuals with elevated basal blood glucose were more susceptible to the adverse metabolic effects of OP exposure. This novel finding has significant implications for the prevention and management of diabetes and metabolic syndrome. It is critical to identify susceptible populations and implement personalized intervention strategies, and individual metabolic status should be considered when developing environmental exposure limits and health risk assessment strategies. However, there are still some limitations. The present study only measured baseline OP concentrations and could not capture dynamic changes in exposure levels. However, the study population mainly consisted of middle-aged and older adults whose lifestyle and dietary habits tend to remain relatively stable over time. Therefore, the baseline serum OP concentrations measured in this study can serve as a reasonable indicator for individual internal exposure. While additional measurements at multiple time points could further strengthen the robustness of our findings, the baseline assessment nonetheless provided meaningful insight into the association between OP exposure and subsequent T2D risk. Moreover, the subjects in the present study were a middle-aged and elderly population; therefore, extrapolation of conclusions to other age groups or populations was restricted. Third, degradation of organophosphorus pesticides is inevitable due to the long storage years of serum samples used in this study. Previous study showed that pesticides such as organophosphorus and pyrethroids were stable at ultra-low temperatures; therefore, the organophosphorus pesticide levels might be relatively stable at −80 °C [48]. Our study suggests that the metabolic effects of organophosphorus pesticide exposure may be modified by baseline glycemic status, while potential influences of gender, lifestyle, and other factors on internal exposure should be considered in interpreting the results. Future studies with larger and more representative populations, longer follow-up periods, and repeated exposure assessments are warranted to better understand the long-term metabolic impact of OPs and refine strategies for metabolic risk assessment.

## 5. Conclusions

No association was observed with T2D risk in the overall population. However, among participants with baseline FBG ≥ 6.1 mmol/L, OP exposure was positively associated with incident T2D and FBG increase during the 5-year follow-up, whereas it was associated with a decrease in FBG in the overall population. A significant interaction with baseline glycemic status suggests that baseline glucose levels may modify the metabolic effects of chronic OP exposure.

## Figures and Tables

**Table 1:** Basic characteristics of the study participants. (N = 2012).

	Serum ΣOrganophosphorus Pesticides Quartiles (ng/L)				*p*
	≤690.7 (N = 503)	690.7–978.1 (N = 503)	978.1–1385.8 (N = 503)	>1385.8 (N = 503)	
Age (years)	62.0 (57.0–67.0)	63.0 (59.0–68.0)	63.0 (58.0–68.0)	63.0 (59.0–67.0)	0.167
Male, N (%)	169 (33.6)	222 (44.1)	243 (48.3)	254 (50.5)	<0.001
Education level, N (%)					0.668
Primary school or below	164 (32.6)	166 (33.0)	157 (31.2)	141 (28.0)	
Middle school	192 (38.2)	182 (36.2)	179 (35.6)	190 (37.8)	
High school	108 (21.5)	113 (22.5)	115 (22.9)	126 (25.0)	
Colleges or universities	39 (7.8)	42 (8.3)	52 (10.3)	46 (9.1)	
Smoking status, N (%)					<0.001
Current smoker	76 (15.1)	91 (18.1)	97 (19.3)	120 (23.9)	
Former smoker	27 (5.4)	59 (11.7)	55 (10.9)	55 (10.9)	
Non-smoker	400 (79.5)	353 (70.2)	351 (69.8)	328 (65.2)	
Drinking status, N (%)					0.001
Current drinker	89 (17.7)	114 (22.7)	114 (22.7)	145 (28.8)	
Former drinker	19 (3.8)	21 (4.2)	31 (6.2)	26 (5.2)	
Non-drinker	395 (78.5)	368 (73.2)	358 (71.2)	332 (66.0)	
Physical activity, N (%)	423 (84.1)	453 (90.1)	453 (90.1)	445 (88.5)	0.009
Shift experience, N (%)	175 (34.8)	178 (35.4)	156 (31.0)	178 (35.4)	0.395
BMI (kg/m^2^)	24.7 (22.5–26.8)	24.6 (22.1–26.9)	24.5 (22.5–26.8)	24.6 (22.0–26.9)	0.904
<18.5, N (%)	8 (1.6)	15 (3.0)	11 (2.2)	11 (2.2)	0.892
18.5~24.0, N (%)	195 (38.8)	199 (39.6)	205 (40.8)	209 (41.6)	
24.0~28.0, N (%)	224 (44.5)	215 (42.7)	205 (40.8)	208 (41.4)	
≥28.0, N (%)	76 (15.1)	74 (14.7)	82 (16.3)	75 (14.9)	
Waist (cm)	83.0 (77.0–89.0)	83.0 (77.0–89.0)	83.0 (77.0–90.0)	82.0 (76.0–89.0)	0.921
Fasting blood glucose (mmol/L)	5.7 (5.3–6.1)	5.7 (5.3–6.2)	5.7 (5.3–6.2)	5.8 (5.4–6.3)	0.039
Total cholesterol (mmol/L)	5.2 (4.6–5.7)	5.1 (4.5–5.8)	5.1 (4.5–5.8)	5.1 (4.6–5.8)	0.997
Triglycerides (mmol/L)	1.2 (0.9–1.7)	1.2 (0.8–1.7)	1.2 (0.9–1.7)	1.2 (0.9–1.8)	0.537
HDLc (mmol/L)	1.4 (1.2–1.7)	1.4 (1.2–1.9)	1.4 (1.2–1.7)	1.4 (1.1–1.6)	0.014
LDLc (mmol/L)	2.9 (2.5–3.4)	2.9 (2.5–3.4)	3.0 (2.5–3.5)	3.0 (2.5–3.6)	0.482
Hypertension, N (%)	220 (43.7)	210 (41.7)	244 (48.5)	223 (44.3)	0.177
Hyperlipidemia, N (%)	228 (45.3)	248 (49.3)	234 (46.5)	233 (46.3)	0.623
Family history of diabetes, N (%)	20 (4.0)	21 (4.2)	12 (2.4)	24 (4.8)	0.235
eGFR (mL/min/1.73 m^2^)	99.2 (87.6–112.8)	96.3 (85.3–111.6)	99.1 (86.5–112.2)	98.1 (86.5–113.3)	0.499

Abbreviation: BMI, body mass index; LDLc, low-density lipoprotein cholesterol; HDLc, high-density lipoprotein cholesterol; eGFR, estimated glomerular filtration rate.

**Table 2:** The detection rates of 29 types of serum organophosphorus pesticides (OPs) in the study participants.

Organophosphorus Pesticides	Participants [Detectable Rate, N (%)]		
	Total Population	Case	Control
Mevinphos	25 (1.24)	11 (1.09)	14 (1.39)
Ethoprophos	934 (46.42)	467 (46.42)	467 (46.42)
Cadusafos	107 (5.32)	51 (5.07)	56 (5.57)
Phorate	865 (42.99)	437 (43.44)	428 (42.54)
Propetamphos	152 (7.55)	78 (7.75)	74 (7.36)
Etrimfos	1526 (75.84)	762 (75.75)	764 (75.94)
Chlorpyrifos-methyl	264 (13.12)	140 (13.92)	124 (12.33)
Malathion	488 (24.25)	250 (24.85)	238 (23.66)
Diazinon	467 (23.21)	231 (22.96)	236 (23.46)
Parathion	1298 (64.51)	635 (63.12)	663 (65.90)
Quinaphos	69 (3.43)	35 (3.48)	34 (3.38)
Paraoxon-methyl	-	-	-
Pirimiphos-methyl	57 (2.83)	25 (2.49)	32 (3.18)
Fenitrothion	1768 (87.87)	886 (88.07)	882 (87.67)
Chlorpyrifos	2009 (99.85)	1004 (99.80)	1005 (99.90)
Fenthion	49 (2.44)	21 (2.09)	28 (2.78)
Pirimiphos-ethyl	39 (1.94)	17 (1.69)	22 (2.19)
Isofenphos	216 (10.74)	103 (10.24)	113 (11.23)
Methidathion	176 (8.75)	87 (8.65)	89 (8.85)
Tetrachlorvinphos	15 (0.75)	7 (0.70)	8 (0.80)
Profenofos	41 (2.04)	21 (2.09)	20 (1.99)
Ethion	113 (5.62)	63 (6.26)	50 (4.97)
Phosmet	-	-	-
Phosalone	32 (1.59)	17 (1.69)	15 (1.49)
Isocarbophos	15 (0.75)	6 (0.60)	9 (0.89)
Fenamiphos	-	-	-
Triazophos	23 (1.14)	9 (0.89)	14 (1.39)
Pyridaphenthion	20 (0.99)	9 (0.89)	11 (1.09)
EPN	3 (0.15)	2 (0.20)	1 (0.10)

**Table 3:** The concentrations of 4 serum organophosphorus pesticides (OPs) with detection rate > 60% in the study participants. (N = 2012).

Organophosphorus Pesticides	Detection Rate	Serum OP Concentration (ng/L)			*p* Value
		Total Population	Case (N = 1006)	Control (N = 1006)	
Etrimfos	75.8%	74.80 (40.91–140.33)	76.43 (40.93–144.08)	73.63 (40.90–137.58)	0.44
Chlorpyrifos	99.9%	95.01 (87.06–112.00)	94.86 (86.83–113.64)	95.32 (87.33–112.29)	0.79
Fenitrothion	87.9%	454.14 (274.41–690.86)	453.80 (274.24–692.51)	455.95 (274.92–684.58)	0.87
Parathion	64.5%	299.72 (141.42–499.14)	298.06 (141.42–504.20)	301.04 (141.42–492.89)	0.60
ΣOrganophosphorus pesticides	-	978.10 (690.70–1385.80)	981.70 (683.10–1396.60)	972.90 (697.10–1382.80)	0.87

Continuous variables with non-normal distribution are represented by P50 (P25–P75), and the Mann–Whitney U test was used. ΣOrganophosphorus pesticides was derived from the addition of 4 OP concentrations.

**Table 4:** Adjusted odds ratio (95% CI) of type 2 diabetes according to quartile of serum organophosphorus pesticides (OPs). (N = 2012).

	Serum OPs Quartiles				*P_trend_*	Linear Model
	Q1	Q2	Q3	Q4		
Etrimfos						
Model 1	1.00 (Ref)	0.96 (0.71, 1.29)	1.06 (0.76, 1.48)	1.18 (0.76, 1.85)	0.40	1.17 (0.95, 1.45)
Model 2	1.00 (Ref)	0.88 (0.61, 1.26)	0.97 (0.65, 1.44)	1.20 (0.70, 2.06)	0.51	1.18 (0.91, 1.53)
Chlorpyrifos						
Model 1	1.00 (Ref)	0.85 (0.65, 1.10)	0.80 (0.59, 1.07)	0.90 (0.66, 1.23)	0.93	0.94 (0.65, 1.38)
Model 2	1.00 (Ref)	0.86 (0.63, 1.19)	0.71 (0.50, 1.02)	0.78 (0.53, 1.13)	0.34	0.89 (0.56, 1.41)
Fenitrothion						
Model 1	1.00 (Ref)	1.00 (0.73, 1.36)	0.96 (0.68, 1.34)	1.03 (0.71, 1.50)	0.98	1.04 (0.88, 1.22)
Model 2	1.00 (Ref)	0.83 (0.57, 1.19)	0.69 (0.46, 1.03)	0.80 (0.51, 1.25)	0.18	0.90 (0.74, 1.09)
Parathion						
Model 1	1.00 (Ref)	0.64 (0.44, 0.93)	0.65 (0.44, 0.95)	0.79 (0.52, 1.19)	0.90	0.85 (0.67, 1.08)
Model 2	1.00 (Ref)	0.72 (0.46, 1.12)	0.73 (0.47, 1.15)	0.96 (0.59, 1.58)	0.93	0.94 (0.71, 1.25)
ΣOrganophosphorus pesticides						
Model 1	1.00 (Ref)	0.83 (0.61, 1.11)	0.92 (0.65, 1.29)	0.96 (0.65, 1.42)	0.99	0.97 (0.71, 1.31)
Model 2	1.00 (Ref)	0.78 (0.55, 1.11)	0.77 (0.52, 1.16)	0.89 (0.56, 1.41)	0.92	0.87 (0.61, 1.24)

*P_trend_* was obtained from the median of each quartile (ln-transformed) in conditional logistic regression models as a continuous variable. Linear model: each ln-transformed concentration was included in the conditional logistic regression model as a continuous variable. Model 1 adjusted for age. Model 2 adjusted for age, BMI, education level, smoking status, drinking status, physical activity, family history of diabetes mellitus, hypertension, and hyperlipidemia.

## Data Availability

Some or all datasets generated and/or analyzed during the current study are not publicly available but are available from the corresponding author on reasonable request.

## Supplementary Materials

The following supporting information can be downloaded at: https://www.mdpi.com/article/10.3390/toxics14040283/s1, Table S1: Detection limits, recovery rate, and precision of serum OPs; Table S2: Stratified analysis based on baseline blood glucose for the association between serum OPs and type 2 diabetes. (N = 2012); Table S3: Stratified analysis of the association between serum Etrimfos and type 2 diabetes. (N = 2012); Table S4: Stratified analysis of the association between serum Chlorpyrifos and type 2 diabetes. (N = 2012); Table S5: Stratified analysis of the association between serum Fenitrothion and type 2 diabetes. (N = 2012); Table S6: Stratified analysis of the association between serum Parathion and type 2 diabetes. (N = 2012); Table S7: Stratified analysis of the association between ΣOrganophosphorus pesticides and type 2 diabetes. (N = 2012); Table S8: Adjusted odds ratio (95% CI) of type 2 diabetes according to serum organophosphorus pesticides (OPs) levels (20% < detection rate < 60%). (N = 2012); Table S9: Stratified analysis of the association between serum Etrimfos and blood glucose changes during 5 years’ follow-up. (N = 2012); Table S10: Stratified analysis of the association between serum Chlorpyrifos and blood glucose changes during 5 years’ follow-up. (N = 2012); Table S11: Stratified analysis of the association between serum Fenitrothion and blood glucose changes during 5 years’ follow-up. (N = 2012); Table S12: Stratified analysis of the association between serum Parathion and blood glucose changes during 5 years’ follow-up. (N = 2012); Table S13: Stratified analysis of the association between ΣOrganophosphorus pesticides and blood glucose changes during 5 years’ follow-up. (N = 2012); Table S14: Association between serum organophosphorus pesticides (OPs) levels and blood glucose changes during 5 years’ follow-up, excluded participants using hypoglycemic drugs. (N = 1830); Table S15: Association of serum organophosphorus pesticides (OPs) levels (20% < detection rate < 60%) with blood glucose changes during 5 years’ follow-up. (N = 2012); Table S16: Association of serum organophosphorus pesticides (OPs) levels (20% < detection rate < 60%) with blood glucose change rate during 5 years’ follow-up. (N = 2012); Table S17: Association between serum organophosphorus pesticides (OPs) and blood glucose at baseline in 2008. (N = 2012); Figure S1: Flowchart of the participants included in the study; Figure S2: Correlation matrix of serum organophosphorus pesticides (OPs) among the case–control study participants; Figure S3: Stratified analysis for the association between serum Etrimfos and blood glucose changes/blood glucose change rate during 5 years’ follow-up; Figure S4: Stratified analysis for the association between serum Chlorpyrifos and blood glucose changes/blood glucose change rate during 5 years’ follow-up; Figure S5: Stratified analysis for the association between serum Fenitrothion and blood glucose changes/blood glucose change rate during 5 years’ follow-up; Figure S6: Stratified analysis for the association between serum Parathion and blood glucose changes/blood glucose change rate during 5 years’ follow-up; Figure S7: Stratified analysis for the association between serum ΣOrganophosphorus pesticides and blood glucose changes/blood glucose change rate during 5 years’ follow-up.
