# Supplementary material for: Association of Serum Organophosphorus Pesticide Levels with T2D Risk and Blood Glucose Changes: A Nested Case–Control Study
Source: Toxics. 2026 Mar 26;14(4):283. doi: 10.3390/toxics14040283 (PMC13119611; doi:10.3390/toxics14040283)
Supplement: Supplementary file 1 [file toxics-14-00283-s001.zip › toxics-4165473-supplementary.pdf]

Supplementary Table S1 Detection limits, recovery rate, and precision of serum OPs.

	LOD	Recovery rate (%)			Intra-batch	Inter-batch
	(ug/L)	0.2ng	2ng	20ng	CV (%)	CV (%)
**Mevinphos**	0.05	10.38	1.02	0.21	109.77	10.39
**Ethoprophos**	0.00	95.88	86.91	112.50	14.91	8.94
**Cadusafos**	0.01	95.49	85.68	101.52	11.40	1.74
**Phorate**	0.04	97.60	83.40	111.01	6.81	10.35
**Propetamphos**	0.02	86.17	92.62	84.67	5.94	7.48
**Etrimfos**	0.04	91.52	81.30	89.63	5.81	1.13
**Chlorpyrifos-methyl**	0.01	80.97	88.85	89.95	8.53	1.79
**Malathion**	0.04	80.87	91.50	92.51	8.73	7.64
**Diazinon**	0.02	87.79	81.85	81.22	4.86	3.47
**Parathion**	0.20	93.21	80.04	80.72	9.64	1.87
**Quinaphos**	0.08	95.14	98.21	85.33	5.14	3.96
**Paraoxon-methyl**	3.00	77.24	15.11	54.30	28.60	9.38
**Pirimiphos-methyl**	0.02	96.93	96.82	84.70	5.03	4.26
**Fenitrothion**	0.05	104.07	89.61	97.16	10.24	5.43
**Chlorpyrifos**	0.01	90.26	96.23	95.70	4.24	1.73
**Fenthion**	0.05	81.51	74.07	74.13	4.70	4.38
**Pirimiphos-ethyl**	0.04	97.20	102.04	87.56	7.09	2.91
**Isofenphos**	0.01	94.33	108.22	87.51	5.30	1.08
**Methidathion**	0.01	91.54	91.80	78.24	11.05	6.66
**Tetrachlorvinphos**	0.10	89.98	68.81	66.30	12.77	6.78
**Profenofos**	0.10	67.08	70.86	50.10	8.80	1.36
**Ethion**	0.02	97.48	101.02	87.25	4.92	3.55
**Phosmet**	0.17	84.30	67.23	55.10	8.22	2.34
**Phosalone**	0.04	77.22	89.26	89.81	14.09	7.60
**Isocarbophos**	0.17	77.26	87.25	90.18	12.02	6.57
**Fenamiphos**	0.83	29.99	4.41	0.30	64.00	15.43
**Triazophos**	0.13	87.64	85.22	71.91	16.15	8.07
**Pyridaphenthion**	0.16	—	—	—	109.77	10.39
**EPN**	0.20	—	—	—	16.82	8.94

LOD: Instruments were analyzed after diluting the low-concentration standard solution with hexane. The concentration of the standard solution corresponding to a signal-to-noise ratio (S/N) of 3 was defined as the instrumental limit of detection.

Recovery rate: Blank controls were prepared with ultrapure water, and three fetal bovine serum samples served as blank matrices. Mixed standards were spiked at three levels (0.2, 2, 20 ng) and subjected to the same pretreatment before analysis. For spike recovery, pretreated serum matrix was spiked with mixed standards and internal standard, then analyzed to calculate recovery rates. Recovery rate(%) = (measured concentration / spiked concentration) × 100

Compounds were considered acceptable for analysis if the recovery rate was between 70% and 120%, intra-batch precision (CV) ≤15% and inter-batch precision (CV) ≤20%.

Supplementary Table S2 Stratified analysis based on baseline blood glucose for the association between serum OPs and type 2 diabetes. (N=2012)

	**Serum OPs quartiles**				P_{trend}	**Linear model**
	**Q1**	**Q2**	**Q3**	**Q4**		
Etrimfos						
GLU<6.1(N=1383)	1.00(Ref)	0.75 (0.54, 1.05)	0.72 (0.52, 1.01)	0.80 (0.57, 1.11)	0.21	0.94 (0.81, 1.08)
GLU⩾6.1(N=629)	1.00(Ref)	1.10 (0.62, 1.96)	1.24 (0.70, 2.19)	1.31 (0.75, 2.31)	0.33	1.14 (0.90, 1.43)
Chlorpyrifos						
GLU<6.1(N=1383)	1.00(Ref)	0.93 (0.67, 1.31)	0.85 (0.61, 1.19)	0.83 (0.59, 1.17)	0.29	0.70 (0.44, 1.10)
GLU⩾6.1(N=629)	1.00(Ref)	0.77 (0.44, 1.33)	0.86 (0.49, 1.51)	1.34 (0.74, 2.43)	0.16	1.63 (0.80, 3.32)
Fenitrothion						
GLU<6.1(N=1383)	1.00(Ref)	0.88 (0.63, 1.23)	0.81 (0.58, 1.13)	0.81 (0.57, 1.14)	0.17	0.97 (0.86, 1.09)
GLU⩾6.1(N=629)	1.00(Ref)	1.24 (0.70, 2.22)	1.37 (0.77, 2.44)	1.28 (0.74, 2.22)	0.29	1.07 (0.88, 1.29)
Parathion						
GLU<6.1(N=1383)	1.00(Ref)	0.87 (0.60, 1.26)	0.91 (0.67, 1.24)	0.86 (0.62, 1.18)	0.34	0.90 (0.75, 1.09)
GLU⩾6.1(N=629)	1.00(Ref)	0.78 (0.42, 1.45)	1.02 (0.60, 1.74)	1.10 (0.65, 1.84)	0.68	1.02 (0.75, 1.38)
ΣOrganophosphorus pesticides						
GLU<6.1(N=1383)	1.00(Ref)	**0.65 (0.47, 0.91)**	0.74 (0.53, 1.03)	0.71 (0.51, 1.00)	0.09	0.88 (0.70, 1.11)
GLU⩾6.1(N=629)	1.00(Ref)	**1.88 (1.06, 3.33)**	**1.79 (1.01, 3.20)**	1.72 (0.99, 2.98)	0.09	1.16 (0.78, 1.71)

GLU represented baseline blood glucose.

P_{trend} was obtained from the median of each quartile (ln-transformed) in logistic regression models as a continuous variable.

Linear model: Each ln-transformed concentration was included in logistic regression model as a continuous variable.

The model was adjusted for age, gender, BMI, education level, smoking status, drinking status, physical activity, family history of diabetes mellitus, hypertension, and hyperlipidemia.

Supplementary Table S3 Stratified analysis of the association between serum Etrimfos and type 2 diabetes. (N=2012)

	Serum Etrimfos quartiles				P_{trend}	Linear model
	Q1	Q2	Q3	Q4		
Gender						
Male (N=888)	1.00(Ref)	0.91 (0.60, 1.38)	1.19 (0.79, 1.79)	1.08 (0.73, 1.61)	0.49	1.08 (0.92, 1.27)
Female (N=1124)	1.00(Ref)	0.91 (0.63, 1.31)	0.79 (0.55, 1.14)	0.95 (0.65, 1.39)	0.70	1.00 (0.85, 1.17)
Smoking Status						
Ever (N=580)	1.00(Ref)	1.03 (0.73, 1.46)	1.13 (0.81, 1.57)	1.16 (0.84, 1.61)	0.31	1.09 (0.96, 1.25)
Never (N=1432)	1.00(Ref)	0.90 (0.73, 1.11)	0.91 (0.74, 1.12)	0.93 (0.76, 1.15)	0.62	0.99 (0.90, 1.08)
Drinking Status						
Ever (N=559)	1.00(Ref)	1.07 (0.74, 1.53)	1.27 (0.91, 1.79)	1.14 (0.81, 1.60)	0.40	1.07 (0.94, 1.23)
Never (N=1453)	1.00(Ref)	0.90 (0.73, 1.10)	0.87 (0.71, 1.07)	0.94 (0.77, 1.16)	0.62	0.99 (0.91, 1.08)
Physical activity						
No (N=238)	1.00(Ref)	0.90 (0.51, 1.58)	1.29 (0.77, 2.15)	0.95 (0.56, 1.63)	0.94	1.02 (0.83, 1.27)
Yes (N=1774)	1.00(Ref)	0.94 (0.78, 1.13)	0.93 (0.77, 1.12)	0.99 (0.82, 1.19)	0.96	1.01 (0.93, 1.09)
HDLc						
Normal (N=1530)	1.00(Ref)	0.89 (0.72, 1.09)	0.93 (0.76, 1.14)	0.99 (0.81, 1.22)	0.92	1.02 (0.94, 1.11)
Abnormal (N=482)	1.00(Ref)	1.09 (0.77, 1.55)	1.08 (0.76, 1.52)	1.03 (0.73, 1.47)	0.94	1.01 (0.87, 1.16)

P_{trend} was obtained from the median of each quartile (ln-transformed) in logistic regression models as a continuous variable.

Linear model: Each ln-transformed concentration was included in logistic regression model as a continuous variable.

The model was adjusted for age, gender, BMI, education level, smoking status, drinking status, physical activity, family history of diabetes mellitus, hypertension, and hyperlipidemia, except for the factor of stratification.

Supplementary Table S4 Stratified analysis of the association between serum Chlorpyrifos and type 2 diabetes. (N=2012)

	Serum Chlorpyrifos quartiles				P_{trend}	Linear model
	Q1	Q2	Q3	Q4		
Gender						
Male (N=888)	1.00(Ref)	1.00 (0.66, 1.51)	0.95 (0.63, 1.43)	1.00 (0.67, 1.49)	0.98	1.26 (0.74, 2.15)
Female (N=1124)	1.00(Ref)	0.86 (0.60, 1.24)	0.83 (0.58, 1.20)	0.91 (0.62, 1.33)	0.76	0.75 (0.46, 1.22)
Smoking Status						
Ever (N=580)	1.00(Ref)	0.99 (0.69, 1.40)	1.13 (0.81, 1.56)	1.06 (0.76, 1.47)	0.69	1.16 (0.77, 1.75)
Never (N=1432)	1.00(Ref)	0.96 (0.79, 1.18)	0.90 (0.73, 1.11)	0.97 (0.79, 1.19)	0.82	0.92 (0.69, 1.23)
Drinking Status						
Ever (N=559)	1.00(Ref)	0.99 (0.70, 1.39)	0.95 (0.67, 1.35)	0.99 (0.71, 1.39)	0.98	1.25 (0.81, 1.93)
Never (N=1453)	1.00(Ref)	0.94 (0.77, 1.15)	0.95 (0.78, 1.17)	0.97 (0.79, 1.19)	0.88	0.91 (0.70, 1.18)
Physical activity						
No (N=238)	1.00(Ref)	0.90 (0.52, 1.57)	0.81 (0.48, 1.38)	1.00 (0.59, 1.68)	0.88	1.11 (0.56, 2.18)
Yes (N=1774)	1.00(Ref)	0.95 (0.79, 1.15)	0.96 (0.80, 1.16)	0.97 (0.80, 1.16)	0.83	0.97 (0.76, 1.24)
HDLc						
Normal (N=1530)	1.00(Ref)	0.96 (0.78, 1.18)	0.93 (0.75, 1.14)	0.99 (0.81, 1.22)	0.95	1.05 (0.79, 1.40)
Abnormal (N=482)	1.00(Ref)	0.95 (0.67, 1.33)	1.02 (0.74, 1.42)	0.94 (0.68, 1.32)	0.79	0.91 (0.63, 1.32)

P_{trend} was obtained from the median of each quartile (ln-transformed) in logistic regression models as a continuous variable.

Linear model: Each ln-transformed concentration was included in logistic regression model as a continuous variable.

The model was adjusted for age, gender, BMI, education level, smoking status, drinking status, physical activity, family history of diabetes mellitus, hypertension, and hyperlipidemia, except for the factor of stratification.

Supplementary Table S5 Stratified analysis of the association between serum Fenitrothion and type 2 diabetes. (N=2012)

	Serum Fenitrothion quartiles				P_{trend}	Linear model
	Q1	Q2	Q3	Q4		
Gender						
Male (N=888)	1.00(Ref)	0.93 (0.61, 1.44)	0.91 (0.60, 1.40)	0.96 (0.63, 1.45)	0.78	1.00 (0.86, 1.16)
Female (N=1124)	1.00(Ref)	0.92 (0.64, 1.32)	0.86 (0.60, 1.25)	1.03 (0.71, 1.49)	0.81	1.00 (0.89, 1.14)
Smoking Status						
Ever (N=580)	1.00(Ref)	0.90 (0.63, 1.30)	1.03 (0.73, 1.47)	1.06 (0.76, 1.49)	0.68	1.02 (0.90, 1.16)
Never (N=1432)	1.00(Ref)	1.00 (0.81, 1.22)	0.90 (0.73, 1.11)	0.97 (0.78, 1.19)	0.54	0.99 (0.92, 1.06)
Drinking Status						
Ever (N=559)	1.00(Ref)	1.22 (0.84, 1.78)	1.29 (0.89, 1.87)	1.32 (0.92, 1.90)	0.12	1.08 (0.94, 1.23)
Never (N=1453)	1.00(Ref)	0.91 (0.75, 1.12)	0.87 (0.71, 1.07)	0.92 (0.75, 1.13)	0.25	0.98 (0.91, 1.05)
Physical activity						
No (N=238)	1.00(Ref)	0.87 (0.53, 1.42)	0.71 (0.41, 1.25)	0.72 (0.43, 1.20)	0.15	0.88 (0.76, 1.03)
Yes (N=1774)	1.00(Ref)	1.00 (0.83, 1.21)	0.99 (0.82, 1.20)	1.04 (0.86, 1.26)	0.76	1.02 (0.96, 1.10)
HDLc						
Normal (N=1530)	1.00(Ref)	1.01 (0.82, 1.24)	1.04 (0.84, 1.28)	1.04 (0.84, 1.29)	0.68	1.02 (0.95, 1.11)
Abnormal (N=482)	1.00(Ref)	0.94 (0.67, 1.30)	0.80 (0.57, 1.12)	0.91 (0.67, 1.24)	0.36	0.96 (0.87, 1.06)

P_{trend} was obtained from the median of each quartile (ln-transformed) in logistic regression models as a continuous variable.

Linear model: Each ln-transformed concentration was included in logistic regression model as a continuous variable.

The model was adjusted for age, gender, BMI, education level, smoking status, drinking status, physical activity, family history of diabetes mellitus, hypertension, and hyperlipidemia, except for the factor of stratification.

Supplementary Table S6 Stratified analysis of the association between serum Parathion and type 2 diabetes. (N=2012)

	Serum Parathion quartiles				P_{trend}	Linear model
	Q1	Q2	Q3	Q4		
Gender						
Male (N=888)	1.00(Ref)	0.71 (0.45, 1.11)	0.86 (0.59, 1.26)	0.92 (0.64, 1.33)	0.71	0.92 (0.74, 1.14)
Female (N=1124)	1.00(Ref)	0.98 (0.65, 1.48)	0.94 (0.67, 1.32)	1.06 (0.74, 1.50)	0.89	1.01 (0.83, 1.24)
Smoking Status						
Ever (N=580)	1.00(Ref)	0.74 (0.51, 1.08)	0.88 (0.64, 1.20)	0.87 (0.65, 1.17)	0.41	0.91 (0.76, 1.09)
Never (N=1432)	1.00(Ref)	1.01 (0.80, 1.27)	0.98 (0.81, 1.19)	1.05 (0.87, 1.27)	0.71	1.02 (0.91, 1.14)
Drinking Status						
Ever (N=559)	1.00(Ref)	0.89 (0.61, 1.31)	1.08 (0.79, 1.48)	1.10 (0.81, 1.49)	0.44	1.04 (0.87, 1.25)
Never (N=1453)	1.00(Ref)	0.93 (0.73, 1.17)	0.92 (0.76, 1.12)	0.96 (0.79, 1.16)	0.55	0.97 (0.86, 1.08)
Physical activity						
No (N=238)	1.00(Ref)	0.86 (0.49, 1.50)	0.78 (0.44, 1.38)	0.97 (0.61, 1.56)	0.73	0.94 (0.71, 1.25)
Yes (N=1774)	1.00(Ref)	0.94 (0.76, 1.16)	0.98 (0.82, 1.16)	1.01 (0.85, 1.20)	0.93	1.00 (0.90, 1.10)
HDLc						
Normal (N=1530)	1.00(Ref)	0.91 (0.72, 1.14)	0.95 (0.78, 1.16)	1.00 (0.83, 1.21)	0.97	0.98 (0.88, 1.10)
Abnormal (N=482)	1.00(Ref)	0.96 (0.64, 1.42)	0.96 (0.70, 1.30)	0.99 (0.73, 1.35)	0.90	0.98 (0.82, 1.18)

P_{trend} was obtained from the median of each quartile (ln-transformed) in logistic regression models as a continuous variable.

Linear model: Each ln-transformed concentration was included in logistic regression model as a continuous variable.

The model was adjusted for age, gender, BMI, education level, smoking status, drinking status, physical activity, family history of diabetes mellitus, hypertension, and hyperlipidemia, except for the factor of stratification.

Supplementary Table S7 Stratified analysis of the association between ΣOrganophosphorus pesticides and type 2 diabetes. (N=2012)

	Serum ΣOrganophosphorus pesticides quartiles				P_{trend}	Linear model
	Q1	Q2	Q3	Q4		
Gender						
Male (N=888)	1.00(Ref)	0.89 (0.58, 1.38)	1.00 (0.66, 1.54)	1.00 (0.65, 1.52)	0.83	1.00 (0.74, 1.36)
Female (N=1124)	1.00(Ref)	0.81 (0.56, 1.16)	0.81 (0.56, 1.17)	0.95 (0.66, 1.38)	0.73	1.00 (0.78, 1.27)
Smoking Status						
Ever (N=580)	1.00(Ref)	1.00 (0.69, 1.44)	1.05 (0.73, 1.50)	1.12 (0.79, 1.57)	0.45	1.04 (0.82, 1.31)
Never (N=1432)	1.00(Ref)	0.93 (0.76, 1.14)	0.91 (0.75, 1.12)	0.94 (0.76, 1.16)	0.52	0.98 (0.86, 1.13)
Drinking Status						
Ever (N=559)	1.00(Ref)	1.08 (0.74, 1.58)	1.30 (0.91, 1.87)	1.22 (0.85, 1.74)	0.21	1.15 (0.90, 1.47)
Never (N=1453)	1.00(Ref)	0.92 (0.75, 1.13)	0.86 (0.70, 1.05)	0.94 (0.76, 1.15)	0.40	0.95 (0.83, 1.10)
Physical activity						
No (N=238)	1.00(Ref)	0.93 (0.57, 1.52)	0.80 (0.47, 1.35)	0.75 (0.44, 1.26)	0.23	0.79 (0.57, 1.10)
Yes (N=1774)	1.00(Ref)	0.95 (0.78, 1.15)	0.98 (0.81, 1.18)	1.03 (0.85, 1.24)	0.71	1.03 (0.91, 1.18)
HDLc						
Normal (N=1530)	1.00(Ref)	0.97 (0.78, 1.19)	1.04 (0.84, 1.28)	1.03 (0.83, 1.28)	0.63	1.03 (0.89, 1.19)
Abnormal (N=482)	1.00(Ref)	0.96 (0.69, 1.33)	0.78 (0.56, 1.09)	0.93 (0.68, 1.27)	0.45	0.94 (0.76, 1.16)

P_{trend} was obtained from the median of each quartile (ln-transformed) in logistic regression models as a continuous variable.

Linear model: Each ln-transformed concentration was included in logistic regression model as a continuous variable.

The model was adjusted for age, gender, BMI, education level, smoking status, drinking status, physical activity, family history of diabetes mellitus, hypertension, and hyperlipidemia, except for the factor of stratification.

Supplementary Table S8 Adjusted odds ratio (95% CI) of type 2 diabetes according to serum organophosphorus pesticides (OPs) levels (20%<detection rate<60%). (N=2012)

**Organophosphorus pesticides**	**Serum OPs levels (detection rate < 60%)**		***P***
	**≤LOD**	**＞LOD**	
**Ethoprophos**	N=1078	N=934	
**Model1**	Ref.	1.00 (0.64, 1.56)	1.00
**Model2**	Ref.	0.91 (0.54, 1.54)	0.72
**Phorate**	N=1147	N=865	
**Model1**	Ref.	1.14 (0.82, 1.57)	0.44
**Model2**	Ref.	0.98 (0.67, 1.44)	0.91
**Diazinon**	N=1545	N=467	
**Model1**	Ref.	0.95 (0.73, 1.23)	0.69
**Model2**	Ref.	0.91 (0.67, 1.25)	0.56
**Malathion**	N=1524	N=488	
**Model1**	Ref.	1.15 (0.83, 1.58)	0.40
**Model2**	Ref.	1.10 (0.76, 1.61)	0.61

Model 1: adjusted for age.

Model 2: adjusted for age, BMI, education level, smoking status, drinking status, physical activity, family history of diabetes mellitus, hypertension, and hyperlipidemia.

Supplementary Table S9 Stratified analysis of the association between serum Etrimfos and blood glucose changes during 5 years' follow-up. (N=2012)

	**Serum Etrimfos quartiles**				P_{trend}	**Linear model**	$P_{interaction}$
	Q1	Q2	Q3	Q4			
**Gender**							0.233
**Male (N=888)**	0.000(Ref)	-0.217 (-0.566, 0.131)	0.021 (-0.319, 0.362)	0.051 (-0.277, 0.380)	0.45	0.071 (-0.063, 0.206)	
**Female (N=1124)**	0.000(Ref)	-0.135 (-0.391, 0.120)	-0.208 (-0.466, 0.050)	-0.141 (-0.409, 0.127)	0.28	-0.037 (-0.149, 0.074)	
**Smoking Status**							
**Ever (N=580)**	0.000(Ref)	-0.340 (-0.780, 0.100)	-0.177 (-0.610, 0.256)	-0.025 (-0.447, 0.397)	0.82	0.053 (-0.122, 0.229)	0.513
**Never (N=1432)**	0.000(Ref)	-0.123 (-0.356, 0.109)	-0.095 (-0.329, 0.138)	-0.068 (-0.306, 0.170)	0.69	-0.003 (-0.102, 0.095)	
**Drinking Status**							
**Ever (N=559)**	0.000(Ref)	-0.231 (-0.631, 0.168)	0.104 (-0.283, 0.491)	0.041 (-0.336, 0.418)	0.50	0.050 (-0.104, 0.204)	0.589
**Never (N=1453)**	0.000(Ref)	-0.158 (-0.402, 0.087)	-0.199 (-0.445, 0.047)	-0.090 (-0.340, 0.160)	0.52	-0.003 (-0.106, 0.101)	
**Physical activity**							
**No (N=238)**	0.000(Ref)	-0.296 (-1.128, 0.536)	0.243 (-0.562, 1.048)	0.073 (-0.716, 0.863)	0.63	0.081 (-0.243, 0.405)	0.717
**Yes (N=1774)**	0.000(Ref)	-0.172 (-0.381, 0.038)	-0.175 (-0.385, 0.035)	-0.073 (-0.285, 0.138)	0.62	0.001 (-0.086, 0.089)	
**HDLc**							
**Normal (N=1530)**	0.000(Ref)	-0.166 (-0.395, 0.063)	-0.153 (-0.383, 0.077)	-0.012 (-0.242, 0.217)	0.94	0.025 (-0.070, 0.120)	0.712
**Abnormal (N=482)**	0.000(Ref)	-0.117 (-0.594, 0.360)	0.000 (-0.472, 0.473)	-0.136 (-0.618, 0.346)	0.68	-0.017 (-0.217, 0.183)	

P_{trend} was obtained from the median of each quartile (ln-transformed) in generalized linear regression model as a continuous variable.

Linear model: Each ln-transformed concentration was included in generalized linear regression model as a continuous variable.

The model was adjusted for age, gender, smoking status, drinking status, education level, BMI, physical activity, family history of diabetes mellitus, and hypoglycemic drugs, except for the factor of stratification.

Supplementary Table S10 Stratified analysis of the association between serum Chlorpyrifos and blood glucose changes during 5 years' follow-up. (N=2012)

	**Serum Chlorpyrifos quartiles**				P_{trend}	**Linear model**	$P_{interaction}$
	Q1	Q2	Q3	Q4			
**Gender**							0.724
**Male (N=888)**	0.000(Ref)	0.072 (-0.278, 0.421)	0.091 (-0.254, 0.436)	0.035 (-0.300, 0.370)	0.97	0.040 (-0.407, 0.487)	
**Female (N=1124)**	0.000(Ref)	-0.003 (-0.258, 0.253)	-0.170 (-0.426, 0.085)	0.052 (-0.215, 0.319)	0.74	0.071 (-0.277, 0.420)	
**Smoking Status**							0.773
**Ever (N=580)**	0.000(Ref)	0.002 (-0.451, 0.455)	0.171 (-0.267, 0.609)	0.118 (-0.314, 0.551)	0.55	0.179 (-0.369, 0.726)	
**Never (N=1432)**	0.000(Ref)	0.031 (-0.201, 0.263)	-0.159 (-0.392, 0.073)	0.009 (-0.228, 0.246)	0.94	0.003 (-0.316, 0.322)	
**Drinking Status**							
**Ever (N=559)**	0.000(Ref)	0.225 (-0.172, 0.622)	0.172 (-0.228, 0.571)	0.078 (-0.307, 0.464)	0.95	0.022 (-0.498, 0.542)	0.762
**Never (N=1453)**	0.000(Ref)	-0.063 (-0.309, 0.183)	-0.151 (-0.394, 0.092)	0.008 (-0.242, 0.258)	0.84	0.054 (-0.273, 0.381)	
**Physical activity**							
**No (N=238)**	0.000(Ref)	0.324 (-0.524, 1.172)	-0.377 (-1.185, 0.431)	-0.212 (-1.007, 0.583)	0.41	-0.064 (-1.147, 1.019)	0.354
**Yes (N=1774)**	0.000(Ref)	-0.032 (-0.242, 0.178)	-0.042 (-0.252, 0.168)	0.053 (-0.159, 0.265)	0.51	0.064 (-0.216, 0.343)	
**HDLc**							
**Normal (N=1530)**	0.000(Ref)	-0.154 (-0.386, 0.077)	-0.174 (-0.406, 0.059)	-0.068 (-0.301, 0.165)	0.9	-0.023 (-0.341, 0.295)	0.514
**Abnormal (N=482)**	0.000(Ref)	**0.507 (0.041, 0.974)**	0.259 (-0.189, 0.706)	0.203 (-0.256, 0.663)	0.83	0.145 (-0.411, 0.702)	

P_{trend} was obtained from the median of each quartile (ln-transformed) in generalized linear regression model as a continuous variable.

Linear model: Each ln-transformed concentration was included in generalized linear regression model as a continuous variable.

The model was adjusted for age, gender, smoking status, drinking status, education level, BMI, physical activity, family history of diabetes mellitus, and hypoglycemic drugs, except for the factor of stratification.

Supplementary Table S11 Stratified analysis of the association between serum Fenitrothion and blood glucose changes during 5 years' follow-up. (N=2012)

	**Serum Fenitrothion quartiles**				P_{trend}	**Linear model**	$P_{interaction}$
	Q1	Q2	Q3	Q4			
**Gender**							0.113
**Male (N=888)**	0.000(Ref)	-0.155 (-0.515, 0.206)	-0.041 (-0.396, 0.314)	-0.028 (-0.378, 0.321)	0.89	0.022 (-0.105, 0.150)	
**Female (N=1124)**	0.000(Ref)	**-0.353 (-0.605, -0.101)**	**-0.328 (-0.585, -0.071)**	-0.226 (-0.485, 0.033)	**0.02**	**-0.086 (-0.171, -0.001)**	
**Smoking Status**							0.304
**Ever (N=580)**	0.000(Ref)	-0.319 (-0.785, 0.148)	-0.086 (-0.553, 0.380)	-0.161 (-0.617, 0.294)	0.58	0.005 (-0.167, 0.177)	
**Never (N=1432)**	0.000(Ref)	**-0.278 (-0.509, -0.047)**	**-0.313 (-0.545, -0.081)**	-0.170 (-0.403, 0.063)	**0.03**	-0.071 (-0.149, 0.006)	
**Drinking Status**							
**Ever (N=559)**	0.000(Ref)	0.010 (-0.404, 0.424)	0.117 (-0.295, 0.528)	0.005 (-0.398, 0.408)	0.85	0.008 (-0.140, 0.156)	0.318
**Never (N=1453)**	0.000(Ref)	**-0.389 (-0.632, -0.146)**	**-0.347 (-0.592, -0.101)**	-0.211 (-0.457, 0.035)	**0.01**	-0.070 (-0.152, 0.013)	
**Physical activity**							
**No (N=238)**	0.000(Ref)	-0.657 (-1.452, 0.137)	-0.191 (-1.025, 0.642)	-0.314 (-1.078, 0.451)	0.35	-0.129 (-0.361, 0.104)	0.215
**Yes (N=1774)**	0.000(Ref)	**-0.242 (-0.455, -0.030)**	-0.207 (-0.419, 0.006)	-0.146 (-0.360, 0.068)	0.09	-0.034 (-0.109, 0.041)	
**HDLc**							
**Normal (N=1530)**	0.000(Ref)	**-0.242 (-0.474, -0.009)**	-0.181 (-0.415, 0.053)	-0.194 (-0.431, 0.044)	0.08	-0.044 (-0.127, 0.039)	0.615
**Abnormal (N=482)**	0.000(Ref)	-0.456 (-0.925, 0.013)	-0.350 (-0.815, 0.115)	-0.099 (-0.537, 0.339)	0.36	-0.066 (-0.212, 0.080)	

P_{trend} was obtained from the median of each quartile (ln-transformed) in generalized linear regression model as a continuous variable.

Linear model: Each ln-transformed concentration was included in generalized linear regression model as a continuous variable.

The model was adjusted for age, gender, smoking status, drinking status, education level, BMI, physical activity, family history of diabetes mellitus, and hypoglycemic drugs, except for the factor of stratification.

Supplementary Table S12 Stratified analysis of the association between serum Parathion and blood glucose changes during 5 years' follow-up. (N=2012)

	**Serum Parathion quartiles**				P_{trend}	**Linear model**	$P_{interaction}$
	Q1	Q2	Q3	Q4			
**Gender**							0.696
**Male (N=888)**	0.000(Ref)	0.010 (-0.368, 0.388)	-0.196 (-0.517, 0.125)	-0.155 (-0.461, 0.151)	0.22	-0.115 (-0.298, 0.068)	
**Female (N=1124)**	0.000(Ref)	-0.171 (-0.457, 0.114)	**-0.427 (-0.664, -0.190)**	-0.184 (-0.430, 0.062)	**0.02**	**-0.158 (-0.299, -0.017)**	
**Smoking Status**							0.303
**Ever (N=580)**	0.000(Ref)	-0.099 (-0.578, 0.380)	**-0.435 (-0.850, -0.021)**	-0.321 (-0.714, 0.071)	0.06	**-0.238 (-0.468, -0.008)**	
**Never (N=1432)**	0.000(Ref)	-0.107 (-0.365, 0.151)	**-0.308 (-0.522, -0.094)**	-0.134 (-0.354, 0.085)	0.06	-0.107 (-0.236, 0.021)	
**Drinking Status**							
**Ever (N=559)**	0.000(Ref)	-0.070 (-0.500, 0.359)	-0.290 (-0.656, 0.076)	-0.090 (-0.439, 0.260)	0.42	-0.130 (-0.339, 0.079)	0.725
**Never (N=1453)**	0.000(Ref)	-0.125 (-0.396, 0.147)	**-0.372 (-0.599, -0.145)**	**-0.242 (-0.473, -0.011)**	**0.01**	**-0.162 (-0.296, -0.028)**	
**Physical activity**							
**No (N=238)**	0.000(Ref)	-0.153 (-0.994, 0.688)	-0.799 (-1.611, 0.012)	0.201 (-0.552, 0.954)	0.89	-0.050 (-0.489, 0.389)	0.429
**Yes (N=1774)**	0.000(Ref)	-0.093 (-0.327, 0.140)	**-0.310 (-0.503, -0.117)**	**-0.231 (-0.426, -0.036)**	**<0.01**	**-0.160 (-0.274, -0.046)**	
**HDLc**							
**Normal (N=1530)**	0.000(Ref)	-0.002 (-0.255, 0.250)	**-0.298 (-0.512, -0.083)**	**-0.233 (-0.448, -0.019)**	**0.01**	**-0.162 (-0.288, -0.035)**	0.776
**Abnormal (N=482)**	0.000(Ref)	-0.452 (-0.982, 0.078)	-0.428 (-0.858, 0.002)	0.028 (-0.404, 0.461)	0.70	-0.072 (-0.320, 0.177)	

P_{trend} was obtained from the median of each quartile (ln-transformed) in generalized linear regression model as a continuous variable.

Linear model: Each ln-transformed concentration was included in generalized linear regression model as a continuous variable.

The model was adjusted for age, gender, smoking status, drinking status, education level, BMI, physical activity, family history of diabetes mellitus, and hypoglycemic drugs, except for the factor of stratification.

Supplementary Table S13 Stratified analysis of the association between ΣOrganophosphorus pesticides and blood glucose changes during 5 years' follow-up. (N=2012)

	**Serum ΣOrganophosphorus pesticides quartiles**				P_{trend}	**Linear model**	$P_{interaction}$
	Q1	Q2	Q3	Q4			
**Gender**							0.140
**Male (N=888)**	0.000(Ref)	0.021 (-0.342, 0.383)	0.046 (-0.309, 0.401)	0.095 (-0.256, 0.447)	0.57	0.041 (-0.211, 0.293)	
**Female (N=1124)**	0.000(Ref)	**-0.343 (-0.592, -0.093)**	**-0.464 (-0.719, -0.209)**	-0.221 (-0.480, 0.038)	**0.04**	**-0.190 (-0.358, -0.022)**	
**Smoking Status**							0.556
**Ever (N=580)**	0.000(Ref)	0.137 (-0.335, 0.609)	0.001 (-0.473, 0.475)	0.071 (-0.384, 0.526)	0.94	-0.038 (-0.356, 0.279)	
**Never (N=1432)**	0.000(Ref)	**-0.340 (-0.569, -0.111)**	**-0.368 (-0.598, -0.138)**	-0.174 (-0.408, 0.060)	0.1	-0.146 (-0.302, 0.010)	
**Drinking Status**							
**Ever (N=559)**	0.000(Ref)	**-0.306 (-0.548, -0.065)**	**-0.377 (-0.621, -0.133)**	-0.141 (-0.390, 0.107)	0.82	-0.021 (-0.303, 0.261)	0.491
**Never (N=1453)**	0.000(Ref)	0.029 (-0.389, 0.446)	0.013 (-0.397, 0.424)	-0.033 (-0.429, 0.364)	0.18	-0.153 (-0.318, 0.012)	
**Physical activity**							
**No (N=238)**	0.000(Ref)	-0.434 (-1.232, 0.364)	-0.265 (-1.072, 0.542)	-0.221 (-1.001, 0.560)	0.62	-0.188 (-0.680, 0.304)	0.555
**Yes (N=1774)**	0.000(Ref)	-0.194 (-0.406, 0.018)	**-0.260 (-0.472, -0.048)**	-0.109 (-0.322, 0.105)	0.28	-0.102 (-0.249, 0.044)	
**HDLc**							
**Normal (N=1530)**	0.000(Ref)	-0.154 (-0.386, 0.078)	-0.187 (-0.420, 0.046)	-0.142 (-0.379, 0.096)	0.24	-0.122 (-0.284, 0.040)	0.971
**Abnormal (N=482)**	0.000(Ref)	-0.380 (-0.846, 0.086)	**-0.495 (-0.956, -0.035)**	-0.025 (-0.465, 0.414)	0.79	-0.085 (-0.380, 0.211)	

P_{trend} was obtained from the median of each quartile (ln-transformed) in generalized linear regression model as a continuous variable.

Linear model: Each ln-transformed concentration was included in generalized linear regression model as a continuous variable.

The model was adjusted for age, gender, smoking status, drinking status, education level, BMI, physical activity, family history of diabetes mellitus, and hypoglycemic drugs, except for the factor of stratification.

Supplementary Table S14 Association between serum organophosphorus pesticides (OPs) levels and blood glucose changes during 5 years' follow-up, excluded participants using hypoglycemic drugs. (N=1830)

		**Serum OPs quartiles**				P_{trend}	**glucose level changes with per ln-OP increase**
		**Q1**	**Q2**	**Q3**	**Q4**		
**Etrimfos**							
	β(95%CI)	0.000 (Ref.)	-0.193 (-0.410, 0.023)	-0.202 (-0.420, 0.015)	-0.066 (-0.284, 0.153)	0.690	0.003 (-0.087, 0.094)
**Chlorpyrifos**							
	β(95%CI)	0.000 (Ref.)	-0.021 (-0.240, 0.198)	-0.116 (-0.334, 0.103)	-0.004 (-0.225, 0.217)	0.982	0.024 (-0.273, 0.321)
**Fenitrothion**							
	β(95%CI)	0.000 (Ref.)	**-0.314 (-0.532, -0.095)**	**-0.247 (-0.467, -0.027)**	**-0.221 (-0.440, -0.003)**	**0.019**	**-0.061 (-0.137, 0.014)**
**Parathion**							
	β(95%CI)	0.000 (Ref.)	-0.155 (-0.392, 0.082)	**-0.390 (-0.591, -0.189)**	**-0.214 (-0.417, -0.011)**	**0.004**	**-0.166 (-0.285, -0.047)**
**ΣOrganophosphorus pesticides**							
	β(95%CI)	0.000 (Ref.)	**-0.264 (-0.482, -0.047)**	**-0.300 (-0.519, -0.082)**	-0.166 (-0.385, 0.054)	0.142	-0.136 (-0.285, 0.013)

P_{trend} was obtained from the median of each quartile (ln-transformed) in generalized linear regression model as a continuous variable.

Adjusted for age, gender, education level, smoking status, drinking status, BMI, physical activity, and family history of diabetes mellitus.

Supplementary Table S15 Association of serum organophosphorus pesticides (OPs) levels (20%<detection rate<60%) with blood glucose changes during 5 years' follow-up. (N=2012)

**Organophosphorus pesticides**	**Serum OPs levels (detection rate < 60%)**		***P***
	**≤LOD**	**> LOD**	
**Ethoprophos**	N=1078	N=934	
**Model1**	Ref.	0.096 (-0.054, 0.245)	0.210
**Model2**	Ref.	0.083 (-0.064, 0.230)	0.270
**Phorate**	N=1147	N=865	
**Model1**	Ref.	**-0.187 (-0.337, -0.037)**	**0.015**
**Model2**	Ref.	**-0.216 (-0.363, -0.068)**	**0.004**
**Diazinon**	N=1545	N=467	
**Model1**	Ref.	**-0.205 (-0.381, -0.029)**	**0.023**
**Model2**	Ref.	**-0.196 (-0.369, -0.023)**	**0.027**
**Malathion**	N=1524	N=488	
**Model1**	Ref.	0.092 (-0.082, 0.267)	0.301
**Model2**	Ref.	0.110 (-0.061, 0.282)	0.208

Model 1: adjusted for age, gender.

Model 2: adjusted for age, gender, BMI, education level, smoking status, drinking status, physical activity, family history of diabetes mellitus and hypoglycemic drugs.

Supplementary Table S16 Association of serum organophosphorus pesticides (OPs) levels (20%<detection rate<60%) with blood glucose change rate during 5 years' follow-up. (N=2012)

**Organophosphorus pesticides**	**Serum OPs levels (detection rate < 60%)**		***P***
	**≤LOD**	**＞LOD**	
**Ethoprophos**	N=1078	N=934	
**Model1**	Ref.	0.096 (-0.054, 0.245)	0.210
**Model2**	Ref.	0.083 (-0.064, 0.230)	0.270
**Phorate**	N=1147	N=865	
**Model1**	Ref.	**-0.187 (-0.337, -0.037)**	**0.015**
**Model2**	Ref.	**-0.216 (-0.363, -0.068)**	**0.004**
**Diazinon**	N=1545	N=467	
**Model1**	Ref.	**-0.205 (-0.381, -0.029)**	**0.023**
**Model2**	Ref.	**-0.196 (-0.369, -0.023)**	**0.027**
**Malathion**	N=1524	N=488	
**Model1**	Ref.	0.092 (-0.082, 0.267)	0.301
**Model2**	Ref.	0.110 (-0.061, 0.282)	0.208

Model 1: adjusted for age, gender.

Model 2: adjusted for age, gender, BMI, education level, smoking status, drinking status, physical activity, family history of diabetes mellitus and hypoglycemic drugs.

Supplementary Table S17 Association between serum organophosphorus pesticides (OPs) and blood glucose at baseline in 2008. (N=2012)

	Serum OPs quartiles				P_{trend}	Linear model
	Q1	Q2	Q3	Q4		
**Etrimfos**						
**Model1**	0.000 (Ref.)	0.018 (-0.058, 0.094)	**0.079 (0.002, 0.155)**	0.073 (-0.004, 0.149)	**0.033**	**0.039 (0.007, 0.071)**
**Model2**	0.000(Ref.)	0.005 (-0.069, 0.079)	0.064 (-0.010, 0.137)	0.066 (-0.008, 0.140)	**0.041**	**0.036 (0.006, 0.067)**
**Chlorpyrifos**						
**Model1**	0.000 (Ref.)	-0.016 (-0.092, 0.061)	-0.036 (-0.112, 0.040)	-0.018 (-0.095, 0.059)	0.706	-0.007 (-0.108, 0.095)
**Model2**	0.000 (Ref.)	-0.006 (-0.080, 0.069)	-0.025 (-0.099, 0.048)	-0.014 (-0.088, 0.061)	0.715	-0.002 (-0.101, 0.096)
**Fenitrothion**						
**Model1**	0.000 (Ref.)	0.029 (-0.047, 0.106)	0.041 (-0.036, 0.117)	**0.122 (0.046, 0.199)**	**0.006**	0.022 (-0.004, 0.048)
**Model2**	0.000 (Ref.)	0.015 (-0.059, 0.089)	0.027 (-0.047, 0.102)	**0.118 (0.043, 0.192)**	**0.009**	0.019 (-0.006, 0.045)
**Parathion**						
**Model1**	0.000 (Ref.)	-0.079 (-0.163, 0.005)	-0.045 (-0.115, 0.026)	0.048 (-0.022, 0.119)	0.343	0.020 (-0.022, 0.061)
**Model2**	0.000 (Ref.)	-0.075 (-0.157, 0.006)	-0.031 (-0.100, 0.037)	0.061 (-0.007, 0.130)	0.154	0.030 (-0.010, 0.070)
**ΣOrganophosphorus pesticides**						
**Model1**	0.000 (Ref.)	0.014 (-0.062, 0.090)	0.030 (-0.047, 0.106)	**0.097 (0.020, 0.174)**	**0.013**	**0.063 (0.011, 0.115)**
**Model2**	0.000 (Ref.)	0.013 (-0.061, 0.087)	0.025 (-0.050, 0.099)	**0.102 (0.027, 0.176)**	**0.008**	**0.067 (0.017, 0.118)**

P_{trend} was obtained from the median of each quartile (ln-transformed) in generalized linear regression model as a continuous variable.

Linear model: Each ln-transformed concentration was included in generalized linear model as a continuous variable.

Model1：Adjusted for age, gender.

Model2：Additionally adjusted for smoking status, drinking status, education level, BMI, physical activity, family history of diabetes mellitus.

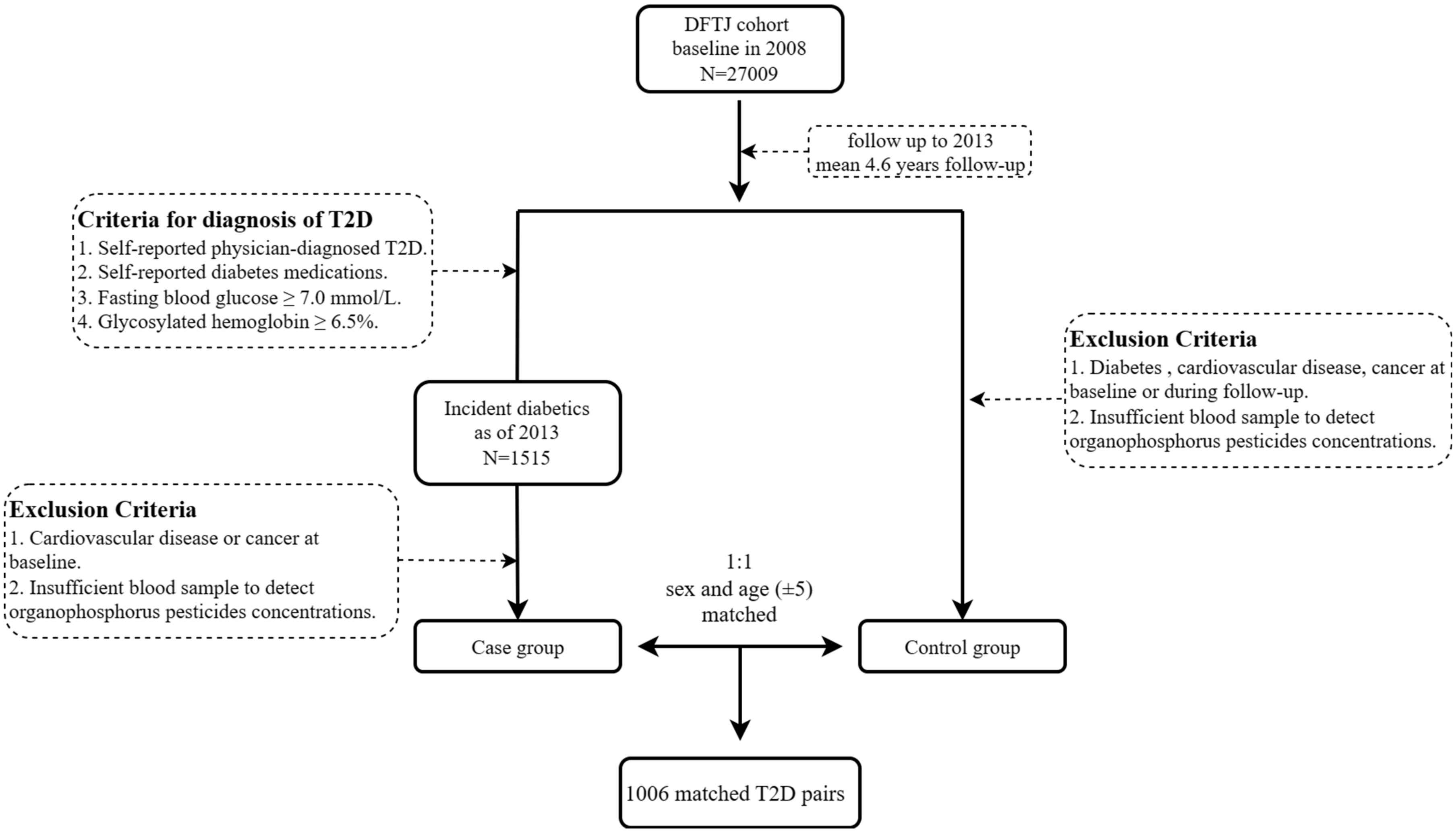

Figure S1 Flowchart of the participants included in the study.

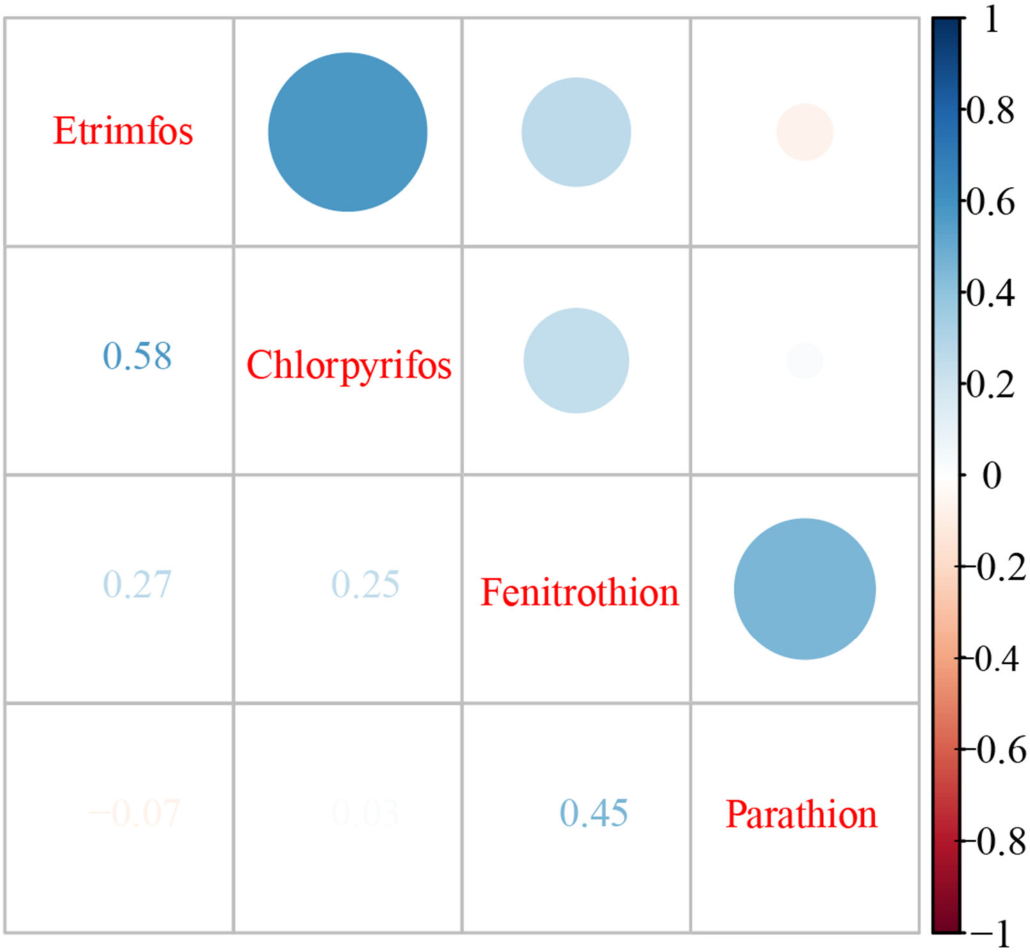

Figure S2 Correlation matrix of serum organophosphorus pesticides (OPs) among the case-control study participants. Spearman's rank correlation coefficients are presented. The size of the dots and the depth of the colour represents the strength of the correlation.

Blood glucose changes

Etrimfos		Linear model	P_{trend}
**Overall**	**(N=2012)**	0.021 (-0.064, 0.107)	
**Q1**			
**Q2**			
**Q3**			
**Q4**			0.982
**Baseline blood glucose<6.1mmol/L**	**(N=1383)**	-0.050 (-0.154, 0.054)	
**Q1**			
**Q2**			
**Q3**			
**Q4**			0.162
**Baseline blood glucose≥6.1mmol/L**	**(N=629)**	**0.197 (0.046, 0.348)**	
**Q1**			
**Q2**			
**Q3**			
**Q4**			**0.022**

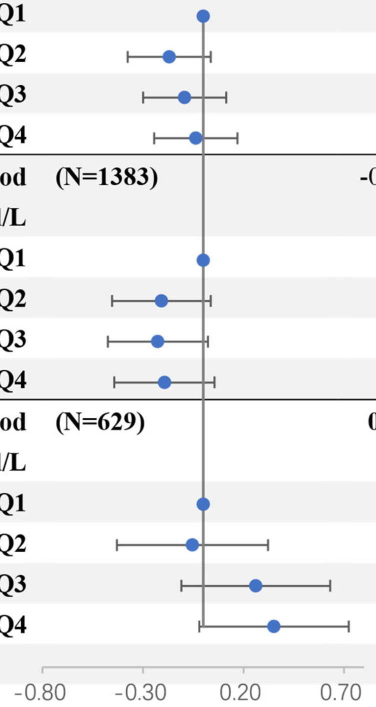

$P_{interaction}$=**0.014**

Blood glucose change rate

Etrimfos		Linear model	P_{trend}
**Overall**	**(N=2012)**	0.000 (-0.016, 0.015)	
**Q1**			
**Q2**			
**Q3**			
**Q4**			0.611
**Baseline blood glucose<6.1mmol/L**	**(N=1383)**	-0.012 (-0.032, 0.008)	
**Q1**			
**Q2**			
**Q3**			
**Q4**			0.116
**Baseline blood glucose≥6.1mmol/L**	**(N=629)**	**0.031 (0.007, 0.054)**	
**Q1**			
**Q2**			
**Q3**			
**Q4**			**0.021**

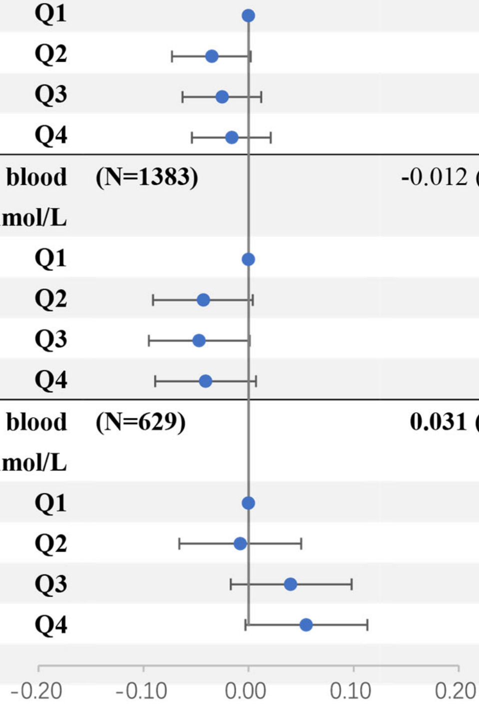

$P_{interaction}$=**0.022**

Figure S3 Stratified analysis for the association between serum Etrimfos and blood glucose changes/ blood glucose change rate during 5 years' follow-up.

P_{trend} was obtained from the median of each quartile (ln-transformed) in generalized linear regression model as a continuous variable.

Linear model: Each ln-transformed concentration was included in generalized linear regression model as a continuous variable.

Adjusted for age, gender, smoking status, drinking status, education level, BMI, physical activity, family history of diabetes mellitus, and hypoglycemic drugs.

Blue dots represented non-significant results, red dots represented statistically significant results.

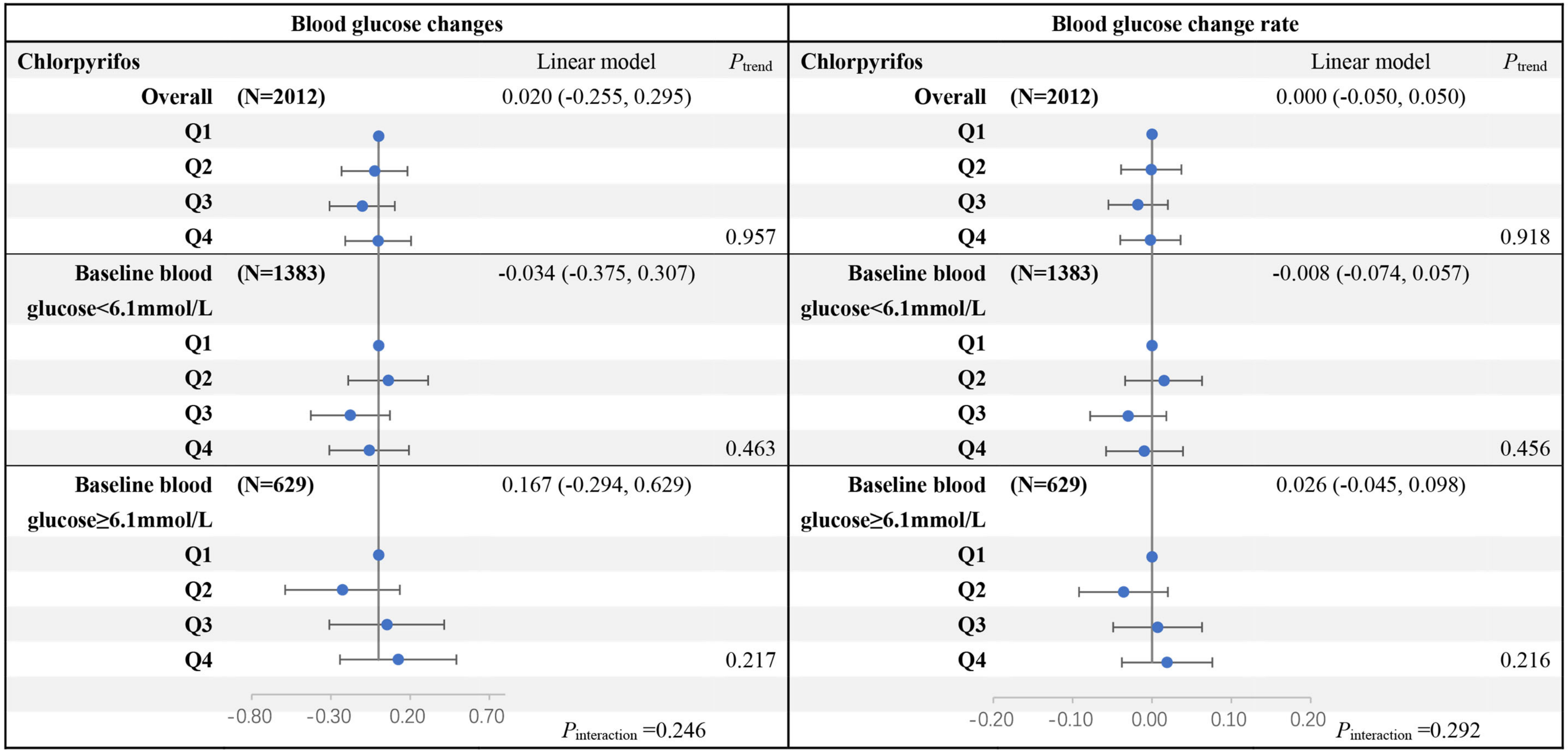

Figure S4 Stratified analysis for the association between serum Chlorpyrifos and blood glucose changes/ blood glucose change rate during 5 years' follow-up.

P_{trend} was obtained from the median of each quartile (ln-transformed) in generalized linear regression model as a continuous variable.

Linear model: Each ln-transformed concentration was included in generalized linear regression model as a continuous variable.

Adjusted for age, gender, smoking status, drinking status, education level, BMI, physical activity, family history of diabetes mellitus, and hypoglycemic drugs.

Blue dots represented non-significant results, red dots represented statistically significant results.

Blood glucose changes

Fenitrothion		Linear model	P_{trend}
**Overall**	**(N=2012)**	-0.054 (-0.125, 0.018)	
**Q1**			
**Q2**			
**Q3**			
**Q4**			**0.041**
**Baseline blood glucose<6.1mmol/L**	**(N=1383)**	-0.083 (-0.169, 0.004)	
**Q1**			
**Q2**			
**Q3**			
**Q4**			**0.003**
**Baseline blood glucose≥6.1mmol/L**	**(N=629)**	0.021 (-0.104, 0.146)	
**Q1**			
**Q2**			
**Q3**			
**Q4**			0.327

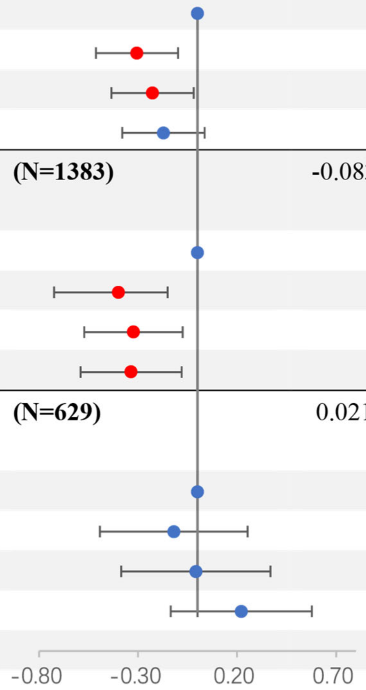

$P_{interaction}$ =0.144

Blood glucose change rate

Fenitrothion		Linear model	P_{trend}
**Overall**	**(N=2012)**	-0.012 (-0.025, 0.001)	
**Q1**			
**Q2**			
**Q3**			
**Q4**			**0.009**
**Baseline blood glucose<6.1mmol/L**	**(N=1383)**	**-0.017 (-0.034, -0.001)**	
**Q1**			
**Q2**			
**Q3**			
**Q4**			**0.001**
**Baseline blood glucose≥6.1mmol/L**	**(N=629)**	0.003 (-0.016, 0.023)	
**Q1**			
**Q2**			
**Q3**			
**Q4**			0.326

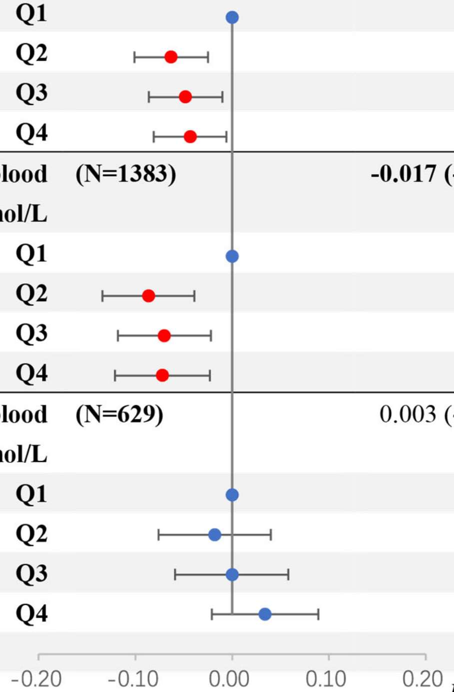

$P_{interaction}$ =0.131

Figure S5 Stratified analysis for the association between serum Fenitrothion and blood glucose changes/ blood glucose change rate during 5 years' follow-up.

P_{trend} was obtained from the median of each quartile (ln-transformed) in generalized linear regression model as a continuous variable.

Linear model: Each ln-transformed concentration was included in generalized linear regression model as a continuous variable.

Adjusted for age, gender, smoking status, drinking status, education level, BMI, physical activity, family history of diabetes mellitus, and hypoglycemic drugs.

Blue dots represented non-significant results, red dots represented statistically significant results.

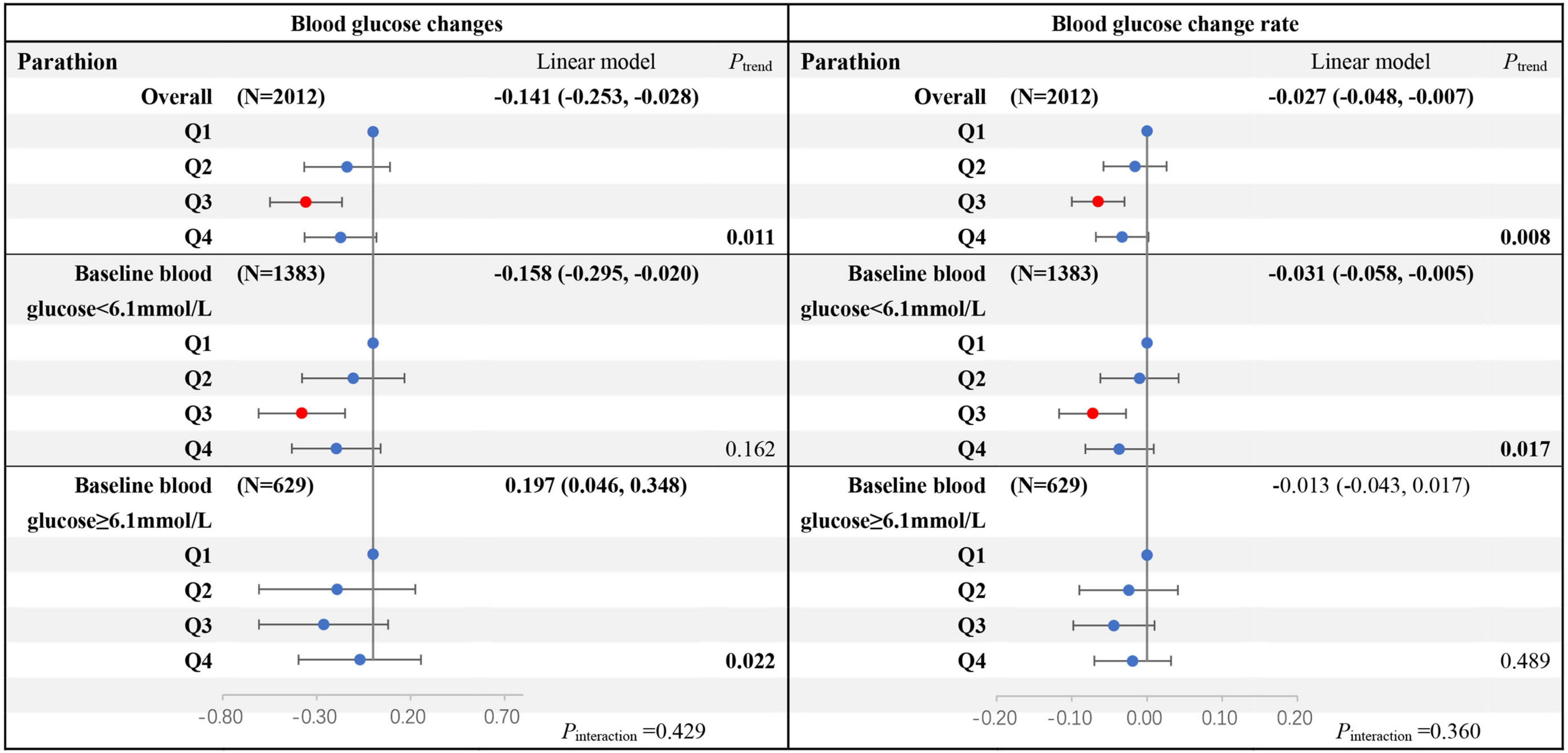

Figure S6 Stratified analysis for the association between serum Parathion and blood glucose changes/ blood glucose change rate during 5 years' follow-up.

P_{trend} was obtained from the median of each quartile (ln-transformed) in generalized linear regression model as a continuous variable.

Linear model: Each ln-transformed concentration was included in generalized linear regression model as a continuous variable.

Adjusted for age, gender, smoking status, drinking status, education level, BMI, physical activity, family history of diabetes mellitus, and hypoglycemic drugs.

Blue dots represented non-significant results, red dots represented statistically significant results.

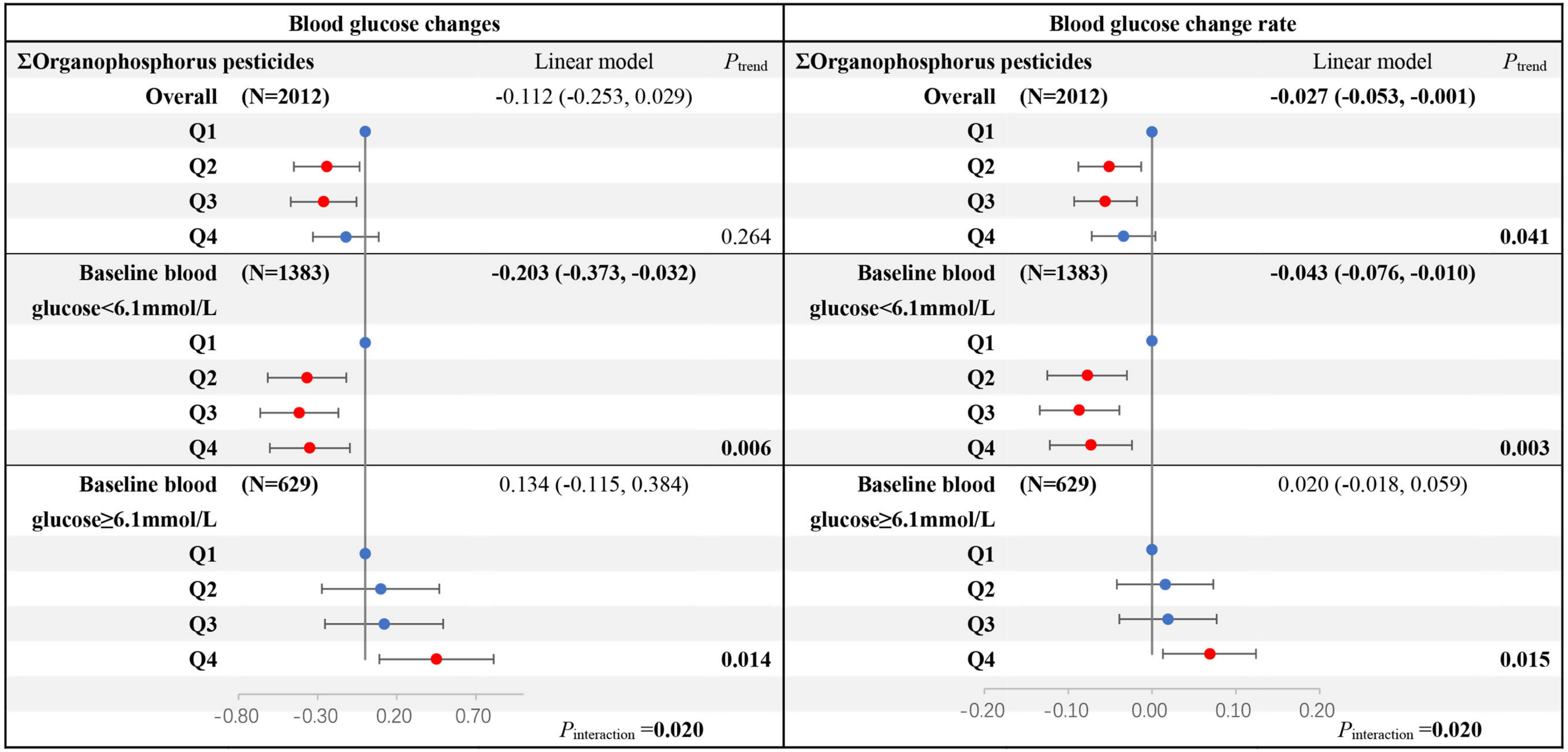

Figure S7 Stratified analysis for the association between serum ΣOrganophosphorus pesticides and blood glucose changes/ blood glucose change rate during 5 years' follow-up.

P_{trend} was obtained from the median of each quartile (ln-transformed) in generalized linear regression model as a continuous variable.

Linear model: Each ln-transformed concentration was included in generalized linear regression model as a continuous variable.

Adjusted for age, gender, smoking status, drinking status, education level, BMI, physical activity, family history of diabetes mellitus, and hypoglycemic drugs.

Blue dots represented non-significant results, red dots represented statistically significant results.
